# Catalytic Pyrolysis of Polyethylene with Microporous and Mesoporous Materials: Assessing Performance and Mechanistic Understanding

**DOI:** 10.1002/cssc.202401141

**Published:** 2024-11-07

**Authors:** Johan H. van de Minkelis, Adrian H. Hergesell, Jan C. van der Waal, Rinke M. Altink, Ina Vollmer, Bert M. Weckhuysen

**Affiliations:** ^1^ Inorganic Chemistry and Catalysis group Institute for Sustainable and Circular Chemistry Utrecht University Universiteitsweg 99 3584 CG Utrecht The Netherlands; ^2^ Brightsite/TNO Urmonderbaan 22 6167 RD Geleen The Netherlands

**Keywords:** chemical recycling of plastics, catalytic pyrolysis, acidity, porosity, zeolites

## Abstract

Testing the catalytic performance for the catalytic pyrolysis of plastic waste is hampered by mass transfer limitations induced by a size mismatch between the catalyst′s pores and the bulky polymer molecules. To investigate this aspect, the catalytic behaviour of a series of microporous and mesoporous materials was assessed in the catalytic pyrolysis of polyethylene (PE). More specifically, a mesoporous material, namely sulfated zirconia (Zr(SO_4_)_2_) on SBA‐15, was synthesized to increase the pore accessibility, which reduces mass transfer limitations and thereby enables to better assess the effect of active site density on catalyst activity. To demonstrate the potential of this approach, the mesoporous SBA‐15 catalysts were compared to a series of microporous zeolite Y catalysts. Using the degradation temperature during thermogravimetric analysis (TGA) as a measure of activity, no correlation between acidity and activity was observed for microporous zeolite Y. However, depending on the *M*_w_ of PE, the reactivity of the mesoporous catalysts increased with increasing Zr(SO_4_)_2_ weight loading, showing that utilizing a mesoporous catalyst can overcome the accessibility limitations at least partially, which was further confirmed by polymer melt infiltration and *in situ* X–ray diffraction. Detailed product analysis revealed that more aromatics and coke deposits were produced with the more acidic zeolite Y materials. The mesoporous material remained active and structurally intact over multiple cycles and catalyses PE degradation via acid‐ and radical‐based pathways.

## Graphical abstract

The pore accessibility and activity have been assessed with a set of microporous and mesoporous catalyst materials for polyethylene pyrolysis. It was found that pore accessibility dominates over acidity for high‐molecular weight polyethylene conversion, thereby showcasing the need to design a new generation of more porous solid acids for the effective catalytic conversion of polyolefin waste.

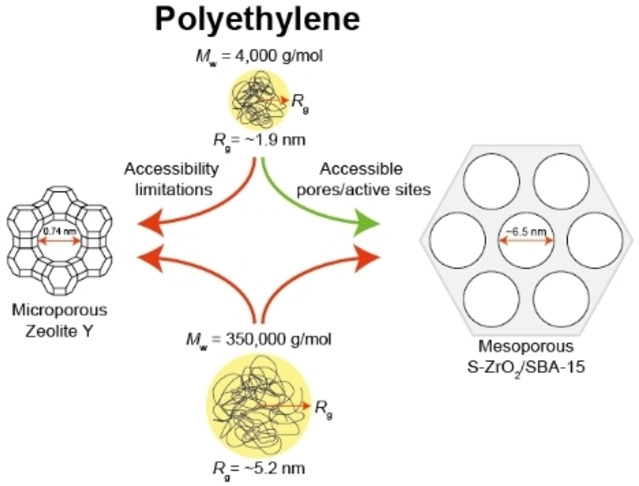

## Introduction

Plastics have become an essential material in our society as they are widely used in many applications, like textiles, electronics, construction, and packaging.^[1]^ This variety of applications is due to the tunable properties of plastics and the high durability of the material.^[2]^ The interest in plastics is still growing, with more than half of all plastic material produced in the last 20 years and with a current annual global production of 400 Mt.^[3]^ The largest part of these plastics is fossil‐based, with only 8.9 % of the annual production based on post‐consumer recycled plastics.^[4]^ From the produced plastics, the biggest share is contributed by commodity plastics, especially polyolefins, like polyethylene (PE, 34 %) and polypropylene (PP, 19 %).^[5]^

Plastic materials can be recycled either mechanically or chemically. Currently, mechanical recycling is the most common recycling technique.^[6]^ A disadvantage of this method is that it yields a material with downgraded properties.^[7]^ To obtain a feedstock that can be used in the production of plastic materials that are close to virgin materials, chemical recycling can be used instead. Techniques, which are currently researched include pyrolysis, hydrocracking, solvolysis, and dissolution/precipitation.^[2]^ It is important to realize that the choice of recycling technique depends on the type of plastic to be recycled. Currently, development of recycling technologies of polyolefins is centered around pyrolysis, because of its versatility and wide feedstock tolerance.^[2]^ With pyrolysis, the polyolefins are decomposed at high temperatures (>400 °C) under an inert atmosphere (N_2_) into a mixture of hydrocarbon gasses, oils and solid chars.^[8]^ However, this thermal decomposition yields low‐value products, like branched and cyclic alkanes.^[9]^ The addition of a catalyst material can lower the temperature of the reaction and increase the activity and selectivity of the pyrolysis reaction towards more value‐added products, like naphtha or aromatic compounds.^[10]^ These products could be used as feedstocks in the production of new plastics or for the synthesis of other bulk and fine chemicals.

For the cracking of the C−C bond of a hydrocarbon, typically solid acids are used, like metal oxides, clays, amorphous silica‐alumina′s, and zeolites (e. g., zeolite β, Y, and ZSM‐5).^[11]^ Zeolite materials consist of a micropore (<2 nm) system with acidic sites introduced by the replacement of Si‐atoms by Al‐atoms in their framework.^[12]^ The acidic sites activate the chemical bonds of hydrocarbons.^[13]^ The acidic sites can be present in two forms, namely as Brønsted acid sites (BAS), introduced by the protonated oxygen atom in the silicon‐alumina framework, or as Lewis acid sites (LAS), introduced by the electron deficient metal atoms in the framework (e. g., Al^*n+*^‐ion expelled from the zeolite framework structure during steaming).^[14]^ Both acid sites are involved in the formation of the carbenium ion as the BAS can donate a proton to an unsaturated hydrocarbon, while the LAS can abstract a hydride from a saturated hydrocarbon.^[15]^ Additionally, the BAS can protonate a saturated hydrocarbon to create a carbonium ion, which can undergo cracking to form a smaller alkane and a new carbenium ion.^[16]^ The produced carbenium ions can subsequently undergo further reactions like β‐scission, isomerization, cyclization and aromatization to form a mixture of different hydrocarbons.^[17]^

While these zeolite‐based materials are widely used in the petrochemical industry, the nature of these materials provides difficulties when used in the conversion of plastics. The microporous systems of the catalyst materials mentioned above introduce severe mass transfer limitations due to the bulky polymers. Polymers cannot reach the active sites that are located inside the zeolite micropores and the utilization of the active sites of traditional catalyst materials is therefore limited.[^18^, ^19^] Additionally, mass transfer limitations make it challenging to investigate inherent catalytic properties in the conversion of plastics.

**Scheme 1:**
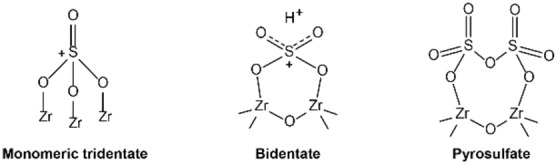
Possible conformations of sulfated zirconia on the surface of a SBA‐15 material after impregnation. Adapted from Rabee et al.^[23]^

Mesoporous materials are a class that could be of interest for the catalytic conversion of plastics. A material that is suitable for this is SBA‐15, which has a highly ordered hexagonal mesoporous (4–30 nm) structure.^[20]^ Besides having mesopores, SBA‐15 is thermally stable, which makes it suitable for the high temperatures applied in the pyrolysis reaction.^[21]^ However, SBA‐15 is a non‐acidic, and thus a non‐catalytic material. To introduce acidic sites, sulfated zirconia (S‐ZrO_2_) was incorporated onto the SBA‐15 material by impregnation with Zr(SO_4_)_2_ ⋅ 4H_2_O salt.^[22]^ After thermally treating the impregnated material, sulfated zirconia can be present as different structures on the surface of SBA‐15. Depending on the loading, the surface species can be present as a monomeric tridentate, a bridging bidentate or a pyrosulfate conformation (Scheme 1).^[23]^ The acidity from these species comes from the sulfur‐hydroxyl groups (BAS) and from the uncoordinated Zr^+^ atoms (LAS).[^24^, ^25^] While a BAS is present in the bidentate conformation, the change towards the pyrosulfate conformation leads to transformation to LAS.^[23]^

The acidic sites introduced by the sulfated zirconia surface species may catalyse the cracking of the hydrocarbon in a similar way as with the more conventional cracking catalyst, based on zeolites. However, there is evidence that sulfated zirconia can catalyse the cleavage of the chemical bonds in hydrocarbons in a different way. For example, Fǎrcaşiu et al. suggests that sulfated zirconia initiates the reaction through a one‐electron oxidation of the hydrocarbon to cation radicals.^[26]^ These are converted towards sulfated esters on the surface and can be transformed to carbocations by ionization or by an elimination of an olefin followed by hydrogenation. This initiates the cracking reaction.

Mesoporous materials have been utilized in the degradation of polyolefins. Aguado et al. studied the use of MCM‐41, Al‐MCM‐41, and Al‐SBA‐15 in the cracking of polyolefin waste and showed that systems with large pores and weak acidity had stronger catalytic activity compared to systems with smaller pores and stronger acidity.[^27^, ^28^] Garforth et al. studied MCM‐41 in HDPE cracking and showed that this material had similar activities to ZSM‐5.^[29]^ Akin et al. studied materials with different pore sizes, HZSM‐5, HZSM‐22, SAPO‐34, SAPO‐11, and Al‐MCM‐41, and showed that materials with larger pores facilitate the formation of aromatics in polyolefin waste pyrolysis.^[30]^ Dai et al. introduced mesoporosity into ZSM‐5 materials, which influenced catalyst lifetime and aromatics selectivity in the cracking of HDPE.^[31]^ Feng et al. studied a tandem porous catalyst consisting of a mesoporous silica shell around a ZSM‐5 material and showed that the shell could crack the bulky polyethylene into intermediates that could diffuse in the ZSM‐5 pores.^[32]^ All these works highlight the importance of large pore systems to overcome the accessibility limitations induced by the bulky plastic molecules and obtain high selectivities towards their desired product fractions. However, a more fundamental understanding of accessibility phenomena is missing, since it is not shown that bulky polymer molecules are indeed able to penetrate the pores and reach the active sites therein.

To showcase the utilization of a mesoporous material in the catalytic depolymerization of PE, thereby removing the accessibility limitations and allowing to investigate intrinsic catalytic properties, we have compared a series of mesoporous materials with a series of microporous materials, more specifically SBA‐15 and zeolite Y, each containing varying amounts of active sites. SBA‐15 was impregnated with an increasing amount of Zr(SO_4_)_2_, which was introduced by impregnation with Zr(SO_4_)_2_ ⋅ 4H_2_O. PE is used as it is hard to decompose due to the difficult to activate C−C bonds, requiring high reaction temperatures, and therefore catalysts are added to lower the temperature and improve selectivity.^[11]^ Additionally, PE makes up the biggest fraction of plastic production and waste, and therefore catalyst should be designed to interact well with this material.^[5]^ By using a mesoporous material, we aimed to probe the active sites without being influenced by mass transfer limitations, that could be induced by the size of the pores (micropores). To study intrinsic activity, while at the same time testing the limits of pore accessibility, we used a PE with a relatively low molecular weight (*M*_w_) of 4,000 g/mol and one with a *M*_w_ of 350,000 g/mol resembling plastic waste, which typically has a *M*_w_ between 100,000 and 500,000 g/mol. By using a multitude of techniques, we showed the intrusion of the polymer material in the mesopores. Additionally, we compared the product selectivity of these catalytic materials in the pyrolysis reaction of both PEs and obtain mechanistic insight on the catalytic pyrolysis using S‐ZrO_2_/SBA‐15 catalyst materials.

## Results and Discussion

SBA‐15 was synthesized by hydrothermal synthesis using PEG‐PPG‐PEG as templating agent.^[33]^ This was followed by impregnation of Zr(SO_4_)_2_ ⋅ 4H_2_O aiming at Zr(SO_4_)_2_ weight loadings of 20, 33, 43, and 50 wt %.^[22]^ These materials are denoted as SZ‐20, SZ‐33, SZ‐43, and SZ‐50. Commercially available zeolite Y materials with different SiO_2_/Al_2_O_3_ ratios of 12, 30, 60, and 80 were chosen, and further denoted as Z–12, Z‐30, Z‐60, and Z‐80, respectively.

### Physicochemical Properties of the Catalyst Materials

**Figure 1:**
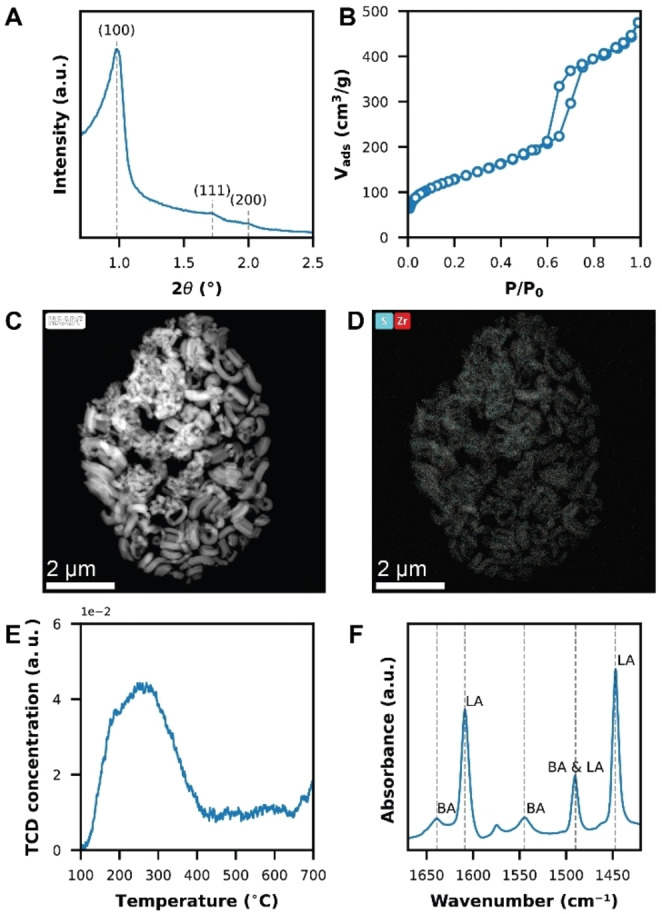
Overview of the material properties of one of the mesoporous S‐ZrO_2_/SBA‐15 catalysts (SZ‐43). (a) X‐ray diffraction (XRD) pattern with the (100), (111), and (200) reflections of SBA‐15 annotated. (b) Isotherm measured by N_2_‐physisorption. (c) High‐angle annular dark field scanning transmission electron microscopy (HAADF‐STEM) and (d) energy‐dispersive X‐ray (EDX) spectroscopy image with elemental mapping of sulfur (blue) and zirconia (red). (e) NH_3_‐Temperature Programmed Desorption (TPD) profile. (f) Fourier transform‐infrared (FT‐IR) spectrum after pyridine adsorption with peak assignment corresponding to Brønsted (BA) and Lewis acid (LA) sites.

The two sets of catalyst materials under study were analysed with X‐ray diffraction (XRD) (Figure 1A) and nitrogen physisorption (Figure 1B) to investigate the structure and porosity of the SBA‐15 material. The results are summarized in Table 1. Detailed information can be found in the Supporting Information (SI 2–5). N_2_–physisorption shows the significant difference in pore sizes between the zeolite Y and S‐ZrO_2_/SBA‐15 materials. The zeolite Y materials have an average pore diameter of 2.8 nm, which is a combination of the regular zeolite micropores of 0.74 nm and some larger mesopores as a result of steaming, while the S‐ZrO_2_/SBA‐15 material showed mesoporosity with an average pore diameter of 6.7 nm. It was found that the BET surface area and pore volume decreased for the S‐ZrO_2_/SBA‐15 materials with an increasing Zr(SO_4_)_2_ weight loading from 648 to 333 m^2^/g and 1.00 to 0.57 cm^3^/g. This suggests that the addition of more catalytic material led to the filling of the SBA‐15 pores and possibly minor pore blockage.

**Table 1:** Physicochemical properties of the synthesized SBA‐15 and the four S‐ZrO_2_/SBA‐15 and zeolite Y catalyst materials under study.

Catalyst material	Lattice spacing d_100_ (nm)^[a]^	Lattice parameter a_0_ (nm)^[b]^	Wall thickness T_w_ (nm)^[c]^	Zirconia crystallite size (nm)	Average pore diameter (nm)^[e]^	BET surface area (m^2^/g) (A)	Pore volume (cm^3^/g) (V)	Number of acid sites (mmol/g)^[f]^	BA:LA ratio^[g]^	Zirconia loading (wt %)^[h]^	Sulfur loading (wt %)^[h]^
SBA‐15	9.05	10.46	4.26	–	6.20	648	1.00	–	–	–	–
SZ‐20	9.55	11.03	4.55	1.42	6.48	468	0.76	0.16	0.18	5.46	0.77
SZ‐33	9.35	10.79	3.87	3.29	6.92	510	0.88	0.19	0.17	7.96	1.11
SZ‐43	9.06	10.46	3.74	3.92	6.72	437	0.74	0.17	0.16	9.95	1.40
SZ‐50	9.15	10.57	3.73	3.98	6.84	333	0.57	0.18	0.21	11.35	1.61
Z‐12	–	–	–	–	2.49	796	0.50	0.73	1.87	–	–
Z‐30	–	–	–	–	2.59	788	0.51	0.44	2.32	–	–
Z‐60	–	–	–	–	2.90	879	0.64	0.23	2.34	–	–
Z‐80	–	–	–	–	3.13	688	0.54	0.21	2.27	–	–

^[a]^ d_100_ calculated from Bragg equation and the (100) reflection in X‐ray diffraction (XRD). ^[b]^ a_0_ = 2d_100_/√3. ^[c]^ T_w_ = a_0_ – average pore diameter. ^[d]^ Average pore diameter = 4 V/A. ^[e]^ Crystallite size calculated from Scherrer equation using the FWHM of the diffraction peak at 2θ 50°. ^[f]^ Determined by NH_3_‐temperature programmed desorption (TPD). ^[g]^ Based on the peak area at 1545 cm^−1^ and 1445 cm^−1^ by Fourier transform‐infrared (FT‐IR) spectroscopy after pyridine adsorption (Py‐FT‐IR). ^[h]^ Determined by inductively coupled plasma‐optical emission spectroscopy (ICP‐OES).

The XRD patterns of the S‐ZrO_2_/SBA‐15 materials at lower 2θ angles showed that the ordered hexagonal structure is present and remained intact after the addition of the Zr(SO_4_)_2_ material, showing the overall structural integrity of the mesoporous materials. This was confirmed by the lattice spacing (d_100_) and parameter (a_0_) and wall thickness (T_w_). Diffractions at 2θ larger than 20° showed the presence of tetragonal zirconia (Figure S3).^[39]^ The intensity of the peaks increased with higher weight loadings. Based on the Scherrer equation and the FWHM of the peak located at 2θ 50°, the zirconia crystallite size showed to grow with higher weight loadings from 1.42 nm to 3.98 nm.

Elemental mapping with high‐angle annular dark field scanning transmission electron microscopy and energy‐dispersive X‐ray spectroscopy (HAADF‐STEM‐EDX) of Zr and S on the SZ‐43 catalytic material shows that the S–ZrO_2_ particles are well distributed over the SBA‐15 crystals without visible aggregates (Figure 1C and D). Elemental analysis with ICP‐OES confirmed that the zirconia and sulfur loading increased with Zr(SO_4_)_2_ loadings used in impregnation.

### Active Species of the Catalyst Materials

We initially hypothesized that increasing amounts of BAS would form on the SBA‐15 catalyst with increasing sulfated zirconia loading and initiate the cracking of PE. Therefore, the catalyst materials were first analysed with ammonia temperature program desorption (NH_3_‐TPD) (Figure 1E) and pyridine Fourier transform infrared spectroscopy (Py‐FT‐IR) (Figure 1F) to investigate the acidic properties. An overview of the acidic properties of the materials is included in Table 1. With NH_3_‐TPD, the total number of acid sites was determined. As expected, the zeolite Y materials show a decrease in total acidity with increasing SiO_2_/Al_2_O_3_, with a higher overall acidity compared to the S—ZrO_2_/SBA—15 materials. However, the S‐ZrO_2_/SBA‐15 materials did have a similar number of total acid sites, independently of the Zr(SO_4_)_2_ weight loading. The decrease in BET surface area and pore volume in N_2_‐physisorption suggest that the mesopores are at least partially blocked at the higher weight loadings, due to particle agglomeration or incomplete dispersion as an effect from the material preparation with incipient wetness impregnation. Therefore, less acidic sites are probed with NH_3_. This discrepancy was also observed by S. Gang et al., who synthesised S‐ZrO_2_/SBA‐15 with varying weight loadings (10–50 wt %).^[34]^ However, this does not explain the result fully as we discuss below. The S‐ZrO_2_/SBA‐15 materials have more LAS, while the zeolite materials are dominated by BAS. Furthermore, the creation of other active species can influence the activity of the S‐ZrO_2_/SBA‐15 materials.

**Figure 2:**
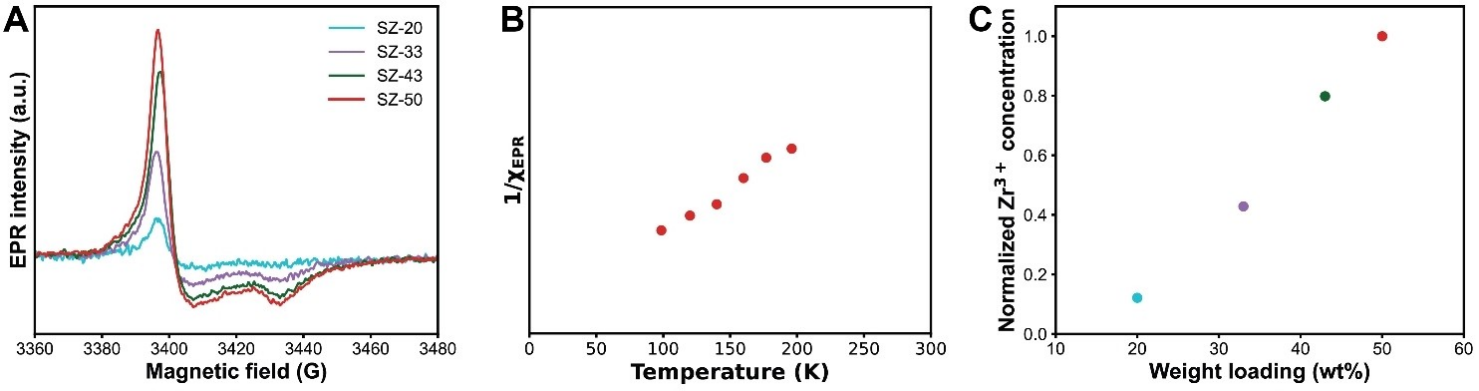
(a) Mass‐normalized electron paramagnetic resonance (EPR) spectra of the four S‐ZrO_2_/SBA‐15 catalyst materials. (b) 1/*χ*_EPR_ vs temperature of the SZ‐50 catalyst material. (c) Normalized Zr^3+^ concentration based on the double integration of the EPR spectra as function of the Zr(SO_4_)_2_ weight loading.

Next to LAS and BAS, Zr^3+^ species can act as active component in S‐ZrO_2_/SBA‐15. The Zr^3+^ species are generated when sulfated zirconia is thermally treated at temperatures higher than 600 °C, resulting in the transformation of SO_4_^2−^ into SO_2_ and O_2_, leading to the reduction of Zr^4+^ into Zr^3+^.[^35^, ^36^, ^37^] To confirm the presence of Zr^3+^, electron paramagnetic resonance (EPR) spectroscopy was applied to observe the unpaired electron of Zr^3+^ (4d^1^ configuration).^[36]^ Figure 2A shows the EPR spectra of the four S—ZrO_2_/SBA‐15 catalyst materials with an axially symmetric powder pattern centered at g_⊥_=1.978 and g_∥_=1.958, which corresponds to a Zr^3+^ ion in a tetragonally distorted environment (D_2d_).^[38]^ Performing EPR at variable temperatures, Curie–Weiss behavior indicative of a paramagnetic material was observed (Figure 2B, S17/18), which is typical for Zr^3+^.[^39^, ^40^] Furthermore, simulation of the Zr^3+^ spectrum showed to fit experimental data well (Figure S19). Comparing the EPR measurements of the different catalysts revealed that the Zr^3+^ concentration increased with increasing Zr(SO_4_)_2_ weight loadings (Figure 2C).

### Pore Accessibility by the Polymer Materials

The radius of gyration (*R*_g_) estimates the size of the polymer coil and can be used to estimate the accessibility into the catalyst material.^[41]^ For one of the investigated PE materials, the low *M*_w_ PE, with a *M*_w_ of 4,000 g/mol and a *M*_n_ of 1,700 g/mol, the radius of gyration based on the *M*_n_ is ~1.9 nm. Compared to the pore diameter of 0.74 nm for the zeolite Y, it means that the polymer coil is larger than the pores and therefore cannot enter them. For the S‐ZrO_2_/SBA‐15 materials with a pore diameter of 6.7 nm, it indicates that the polymer can access the active sites inside the pores of the mesoporous support.

For the other polymer material under study, the high *M*_w_ PE, with a *M*_w_ of 350,000 g/mol and a *M*_n_ of 13,000 g/mol, the radius of gyration is ~5.2 nm. This value is substantially larger than that for the low *M*_w_ PE and exceeds the pore diameter of both porous materials under investigation, indicating accessibility limitations.

To study the pore accessibility of the polymer materials, we monitor the intrusion of the polymer into the pores with *in situ* XRD. A 2 : 1 PE‐to‐SBA‐15 mixture was heated to 150 °C under N_2_, which is above the melting point of PE (130 °C), and subsequently cooled to room temperature. The scattering at low angles is based on the changes in electron density by the periodic pore structure in the nanometer range.[^42^, ^43^] If the polymer material enters the mesopores of SBA‐15, the intensity of the (100) reflection of SBA‐15 (Figure 1A) is expected to decrease, as the long‐range diffraction of the periodic pore structure is disturbed. The reduction of scattering at low angles by introducing different materials into ordered porous materials is known.[^44^, ^45^] If the polymer is too large to enter the mesopores, the (100) reflection of SBA‐15 would not be influenced.

**Figure 3:**
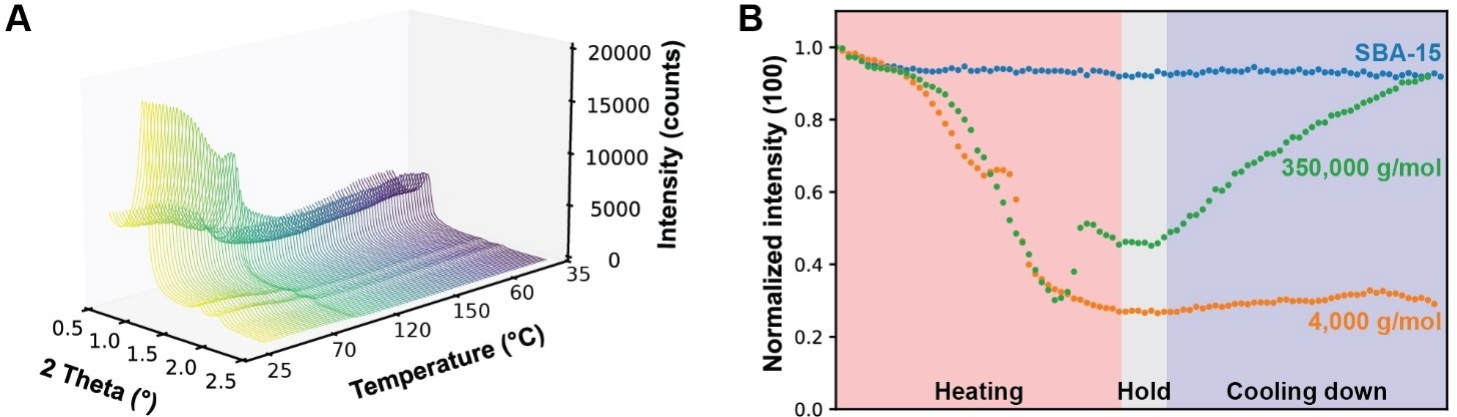
(a) A set of *in situ* X‐ray diffraction (XRD) patterns of melting polyethylene (PE) with a *M*_w_ of 4,000 g/mol in SBA‐15 obtained upon heating to 150 °C and subsequent cooling down to 35 °C in N_2_. (b) Intensity of the SBA‐15 (100) reflection during the *in situ* XRD measurements for the melting of the two different *M*_w_ PEs (4,000 and 350,000 g/mol) in the SBA‐15 material and cooling them down to 35 °C.

Using SBA‐15 without any polymer material, it was observed that the intensity of the (100) reflection remained at similar values when the temperature increased (Figure 3B). This shows that the mesoporous structure is not affected by the temperature increase. When introducing the polymer material to the SBA‐15, the intensity of the (100) peak decreased upon melting of low *M*_w_ PE (Figure 3A and B). This suggests that the polymer is able to enter the pores when molten, and remains there after cooling down. This is in accordance with the radius of gyration that is smaller than the pore size of the SBA‐15 material.

However, when using high *M*_w_ PE, the intensity of the (100) reflection decreased when heating, but increased again after cooling down to an intensity value similar to the initial state. This suggests that the polymer is partially able to access the mesopores, but upon slowly cooling down, the polymer is leaving the mesopores to restore the (100) reflection of the SBA‐15 material. As the comparison of radius of gyration with pore size suggests, the polymer is not able to enter as a coil, but first has to untangle. Partially untangled polymer chain ends might be able to thread into the pore, but upon slow cooling, the polymer coils up again and the polymer chain ends retracts from the mesopores.

### Catalytic Performance

To evaluate the catalytic activity, the two catalyst materials were combined with polyethylene and subjected to thermogravimetric analysis (TGA). Herein, the materials are heated under a nitrogen flow, to decompose the polymer material. Once this occurs, the weight of the sample decreases, and this is represented in the TGA profile. The derivative of the weight loss (DTGA) can be seen as a measure of catalytic performance, with a shift towards lower temperatures indicating a higher catalytic activity. The catalyst materials were assessed with low *M*_w_ and high *M*_w_ PE.

**Figure 4:**
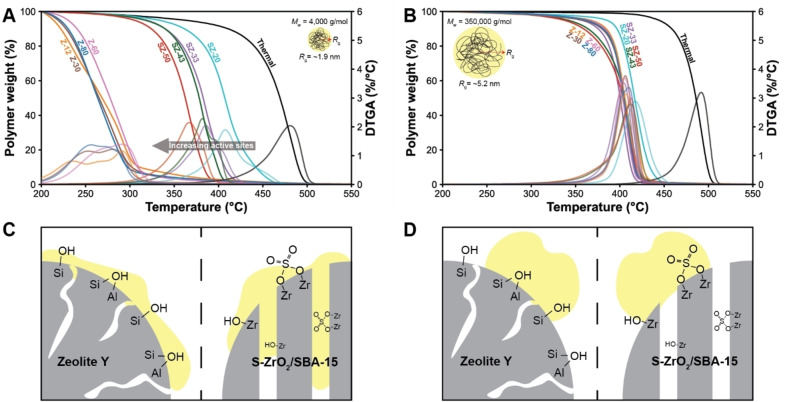
Thermogravimetric analysis (TGA) profiles with their derivatives (DTGA) of the four zeolite Y (Z) and S–ZrO_2_/SBA‐15 (SZ) catalyst materials under study with (a) low (4,000 g/mol) and (b) high (350,000 g/mol) *M*_w_ polyethylene (PE). A schematic representation of the PE coil sizes based on *R*_g_ is presented in both figures. Schematic representation of the PE‐catalyst material interaction for (c) low *M*_w_ and (d) high *M*_w_ PE with a zeolite Y and a S‐ZrO_2_/SBA‐15 catalyst particle, based on the *in situ* XRD measurements, the radius of gyration calculations and TGA for both PEs and catalyst materials.

Without the addition of a catalyst material, the decomposition of low *M*_w_ PE occurs at ~483 °C. For the microporous zeolite Y materials, the decomposition temperature is lowered by ~200 °C (Figure 4A). However, no dependency on the acidity of the catalyst materials was observed. As discussed in a previous section, the acidity decreases with increasing SiO_2_/Al_2_O_3_. It was expected that the catalytic activity would scale with the acidity of the catalytic material. That no activity trend was observed with the acidity suggests that the zeolite material is limited by accessibility, which is in correspondence with literature and also expected based on the radius of gyration of this polymer material.[^18^, ^19^] This result shows that using the microporous zeolite Y materials prevents us from assessing the inherent effect of active site density and type on activity.

However, the accessibility limitations can be overcome with the mesoporous S‐ZrO_2_/SBA‐15 materials. With the low *M*_w_ PE, a decrease in decomposition temperature by 70 to 120 °C was observed with an increasing Zr(SO_4_)_2_ weight loading. This indicates that the mesoporous supports can be used to assess the intrinsic activity of the catalyst material. However, the observed trend does not match the acidity of the catalyst materials under study, as NH_3_‐TPD measurements showed that all the mesoporous materials had similar acidity. Therefore, the degradation temperature cannot be coupled directly to the acidity of the mesoporous materials but only to the amount of active Zr(SO_4_)_2_ added. This shows that a different component of the material is responsible for the activity observed. The presence of Zr^3+^ species could be related to the increased activity, as literature showed that these species can act as radical initiator and thereby can form hydrocarbon radicals.[^26^, ^35^, ^38^] These species can undergo free radical decomposition and form smaller hydrocarbons. In a previous section it was shown that the Zr^3+^ concentration increased with Zr(SO_4_)_2_ weight loading, and therefore the observed difference in activity in TGA is potentially driven by the redox behavior of sulfated zirconia instead of the acidity.

While the catalyst materials decreased the degradation temperature of high *M_w_* PE (Figure 4B), all the porous materials show a similar degradation temperature independent of acidity or pore size. This indicates that the polymer molecules are too large to access the pores of both the microporous zeolite Y as well as the mesoporous SBA‐15 material and thus do not interact with the active sites located inside the pores. This shows that in combination with the high *M*_w_ PE, the SBA‐15/S‐ZrO_2_ cannot be used to assess the intrinsic activity.

Based on the radius of gyration, *in situ* XRD, and the TGA results, we can hypothesize about the interactions between the two types of PE and the different catalyst materials studied. We conclude that the low *M*_w_ PE can intrude the mesopores of the SBA‐15, based on the decrease in the intensity of the (100) reflection. In addition, the observed trend in activity suggests that the polymer can interact with the active sites. For the microporous materials, we propose that the low *M*_w_ PE flows over the catalyst particle due to the low melt viscosity and mostly interacts with the active sites on the outer surface of the catalyst particles (Figure 4C). In contrast, the high *M*_w_ PE cannot enter the pores of neither the microporous nor mesoporous materials, evident from the lack of a trend of activity with number of active sites. Based on previous studies in our group, it is proposed that the high *M*_w_ polymer does not flow over the surface and reach the full outer surface area of both porous materials due to the higher melt viscosity induced by the increased *M*_w_ of the polymer, which explains the similar activity of both materials as observed with TGA.^[18]^ Only after some pre‐cracking of the polymer on the outer surface of the catalyst particle, smaller hydrocarbons are formed which can enter the pores of both materials where the hydrocarbons can interact with the active sites (Figure 4D).

**Figure 5:**
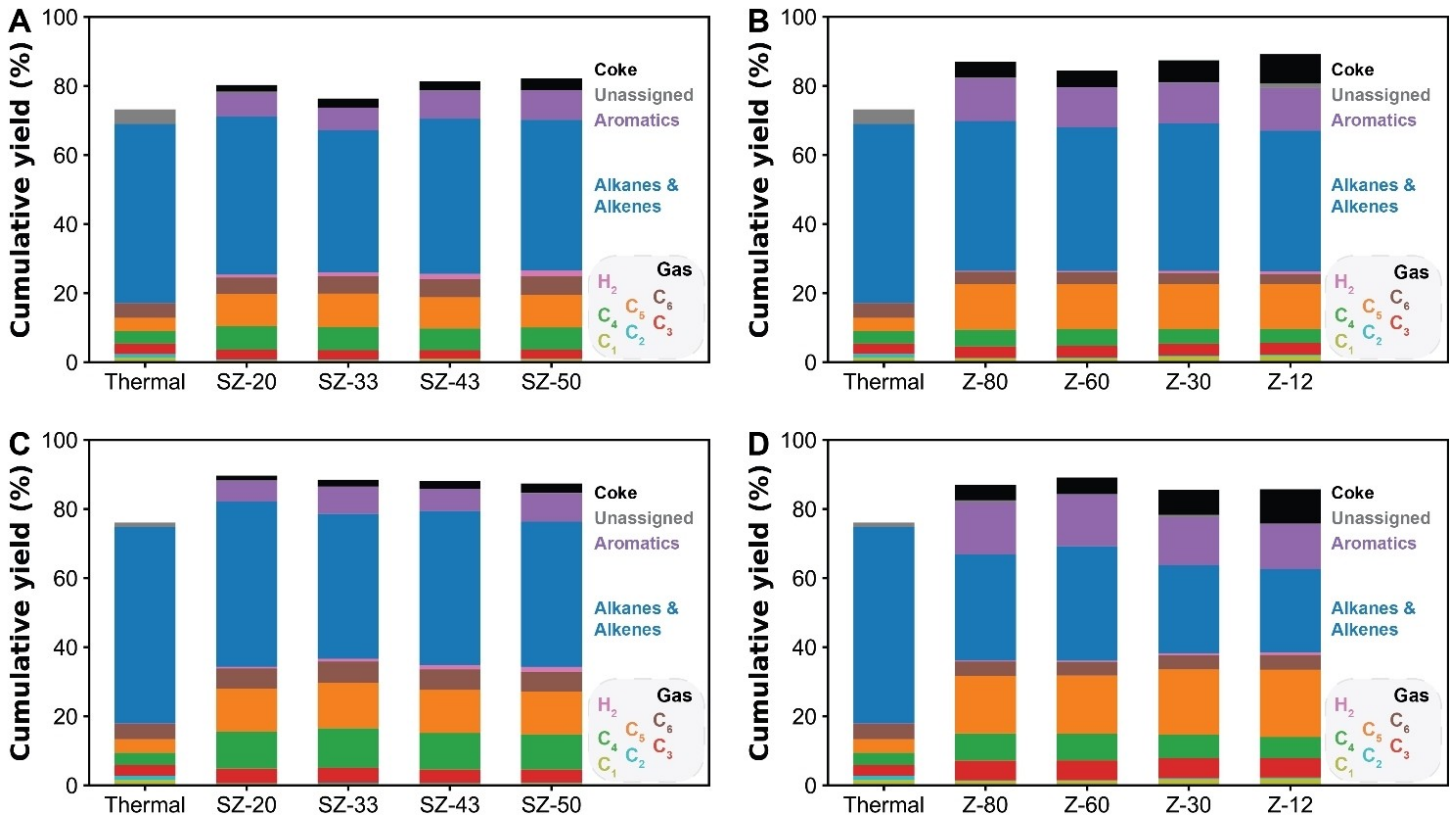
Product distributions of the pyrolysis reaction of low *M*_w_ polyethylene (PE) (4,000 g/mol) with (a) S‐ZrO_2_/SBA‐15 and (b) zeolite Y catalyst materials and high *M*_w_ PE (350,000 g/mol) with (c) S‐ZrO_2_/SBA‐15 and (d) zeolite Y catalyst materials under study.

Besides catalyst activity, the product selectivity is also of interest. To analyse this in a proper manner, the microporous and mesoporous catalyst materials were used in the catalytic pyrolysis of the different PEs using a semi‐batch reactor on the 1 g PE scale at 400 °C. The product distribution in the gas phase was determined by online‐gas chromatography (GC) coupled with mass spectrometry (MS), the liquid phase was collected and analysed off‐line using two dimensional GC (GCxGC). By measuring the gas phase online, it was possible to follow the product formation during the pyrolysis reaction. Including the coke analysis of the spent catalyst materials with the TGA method, we were able to determine the overall product distribution of the pyrolysis reaction and close the mass balance to within 80 % for the non‐catalytic reaction and about 90 % for the catalytic reaction. This distribution is given in Figure 5.

Compared to non‐catalytic pyrolysis, reaction with catalyst material leads to higher total gas yields, with the highest gas yield observed for the microporous zeolite Y materials with both PEs. Additionally, the distribution of products in the gas phase shifted towards longer hydrocarbons, with the highest selectivity towards C_6_ hydrocarbons, and a decrease in C_1_ and C_2_ hydrocarbons. Compared to the zeolite Y materials, the S‐ZrO_2_/SBA‐15 catalyst produced 1–2 wt % less gaseous compounds, with a lower selectivity towards the smallest C_1_ and C_2_ hydrocarbons and more towards C_4_ and C_5_ hydrocarbons, for both *M*_w_ PEs.

**Figure 6:**
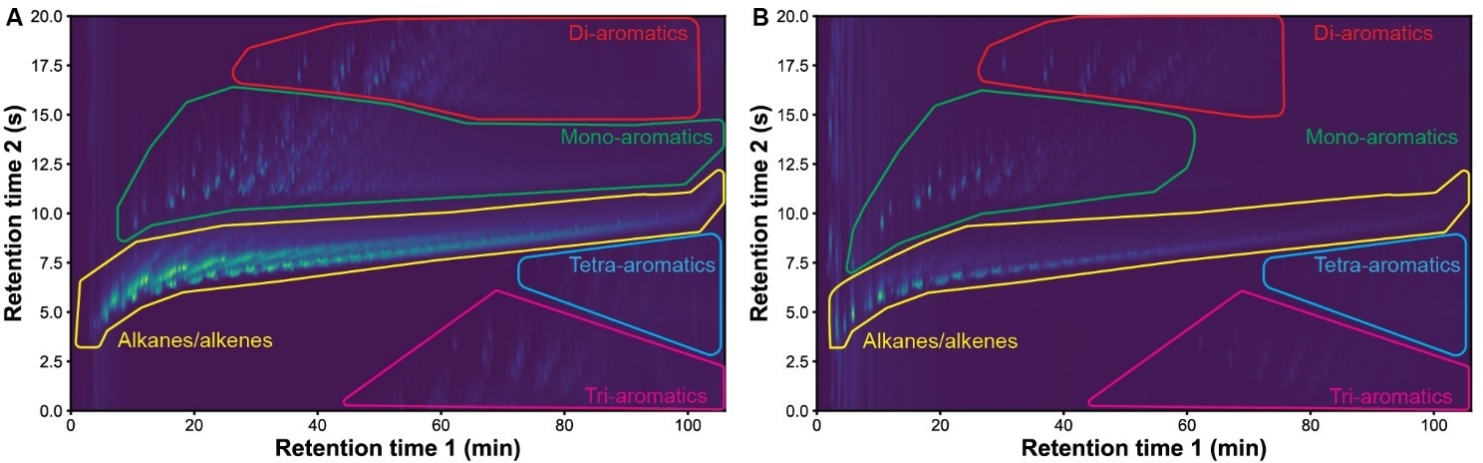
GCxGC chromatograms of the liquid products from the pyrolysis reaction of low *M*_w_ polyethylene (PE) with SZ‐50 (a) and Z‐12 (b) as comparison between the two different materials. Compounds are separated on two columns, with the first separating on boiling point (retention time 1) and the second separating on polarity (retention time 2) The hydrocarbon types are indicated by five different assignments.

The production of a mixture of liquid hydrocarbons was higher for the pyrolysis with S‐ZrO_2_/SBA‐15 compared to the reaction with zeolite Y. This was especially the case for the high *M*_w_ PE, producing between 49 and 53 wt % with S‐ZrO_2_/SBA‐15 while over zeolite Y between 34 and 38 wt % was formed. The oil products were analysed by GCxGC and grouped by alkanes/alkenes and aromatic compounds (Figure 6). A minor part of the GC chromatogram could not be identified and is labelled as unassigned. Alkanes/alkenes are the major products for all catalyst materials and PEs. Reactions using S‐ZrO_2_/SBA‐15 produced more of alkanes/alkenes (41–57 wt %) compared to the zeolite Y materials (34–43 wt %). The zeolite Y materials were found to be more selective towards aromatics (11–15 wt %) compared to S‐ZrO_2_/SBA‐15 materials (6–8 wt %).

The differences in reaction products are attributed to the different acidities of the catalyst materials. The higher acidity of the zeolite Y materials makes them more reactive and there is a higher chance of overcracking. This is why a higher gas yield is obtained with the zeolite Y materials. Additionally, the differences in the liquid hydrocarbons can be assigned to this phenomenon. The polymer chain is first subjected to β‐scission, resulting in unsaturated hydrocarbons. Consecutive reactions could result in isomerisation of the hydrocarbons and cyclization of unsaturated hydrocarbons followed by dehydrogenation to aromatic compounds.^[38]^ With higher acidity, the hydrocarbons will undergo more aromatization and therefore are more prone to coking. In addition, a difference is observed in the type of aromatic compounds. With zeolite Y, mono‐aromatics, like toluene, xylene and mesitylene at lower retention times were observed (Figure 6B). However, while these mono‐aromatic compounds were also present for the S‐ZrO_2_/SBA‐15 materials, more mono‐aromatic compounds with longer or more branched side chains are observed, at higher retention times on first separation (Figure 6A). The larger molecules can be formed because the larger pores of the S‐ZrO_2_/SBA‐15 compared to zeolite Y. The micropores of the zeolite Y favours mono‐aromatics with less branching through shape selectivity.

The final product in the pyrolysis of PE is the coke deposited on the catalyst materials. For non‐catalytic pyrolysis, no coke deposits were observed in the autoclave reactor for both PEs. The highest coke yield was observed for the zeolite Y materials. With low *M*_w_ PE, zeolite Y produced 5 to 8 wt %, while with high *M*_w_ PE it produced 4 to 10 wt % coke. The coke formation increased with increasing acidity of the catalyst material. The S‐ZrO_2_/SBA‐15 materials, due to their lower acidity, produces significantly less coke, with 2 to 3 wt % and 1 to 3 wt % for low and high *M*_w_ PE respectively. For these catalyst materials, the increase in coke formation was correlated to the Zr(SO_4_)_2_ weight loading on the materials. As mentioned before, the higher acidity increases the activity, and therefore the hydrocarbons are more prone towards coking. Besides this, the different pore sizes have an influence on the rate of coke formation. The smaller size of the micropores of the zeolite limits the transport of the cracked hydrocarbons, and therefore make them more prone to subsequent reactions as they have a longer residence time at the active sites. This increases the chance of coke formation, while the larger size of the mesopores enable the transport of the hydrocarbons out of the pores and therefore remove them from the active sites.

**Figure 7:**
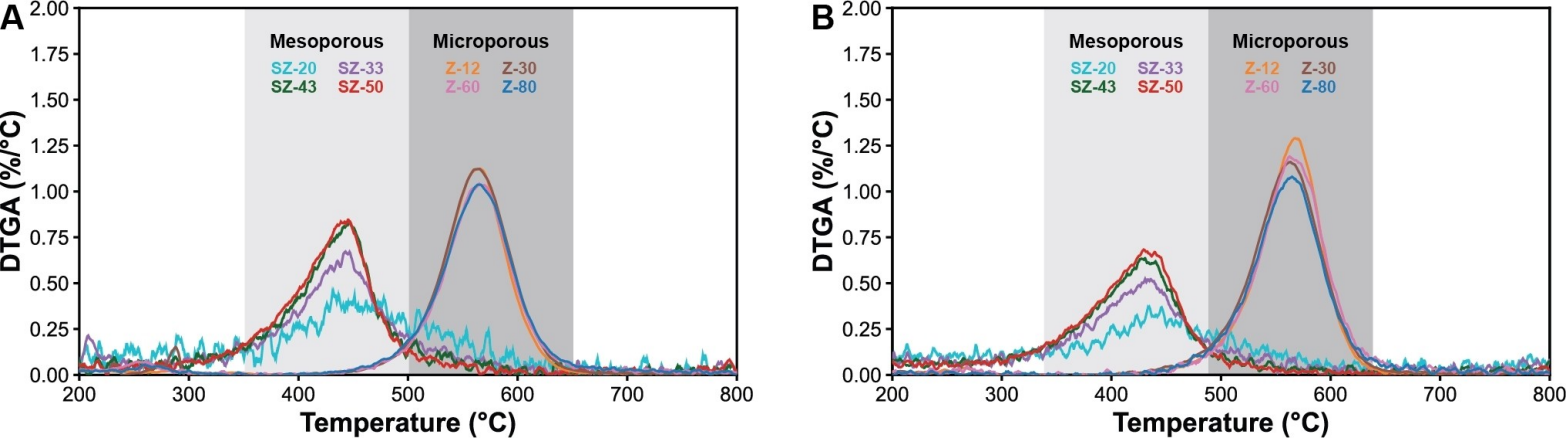
Coke burn‐off by thermogravimetric analysis (TGA) of the spent catalyst materials after pyrolysis with (a) low (4,000 g/mol) and (b) high (350,000 g/mol) *M*_w_ PE.

Additionally, the burn‐off temperature at which the coke deposits could be removed were lower for the S‐ZrO_2_/SBA‐15 catalyst compared to the zeolite Y materials. These were ~440 °C and ~580 °C respectively in the case of both low and high *M*_w_ PE, as can be observed in Figure 7. The difference in burn‐off temperature can be explained by the difference in coke species formed on the two catalyst materials that require different temperatures to decompose. The type of aromatics found in the liquid phase could give an indication of this, as the liquid phase produced by the zeolite Y materials consist of more tri‐ and tetra‐aromatics compared to the aromatics formed by S‐ZrO_2_/SBA‐15. These tri‐ and tetra‐aromatics are the precursors of the coke deposits and therefore show that the coke species on the zeolite Y materials consist of more complex, larger, graphitic materials. Additionally, the difference in pore size between the two catalyst materials could also influence the removal of coke species, as the diffusion of O_2_ is easier for the mesoporous material.

### Catalyst Stability

Given the promising catalytic pyrolysis performance data obtained with the sulfated zirconium‐containing SBA‐15 materials, it is important to further assess their thermal stability. The four S–ZrO_2_/SBA‐15 materials were investigated with thermogravimetric analysis coupled with mass spectrometry (TGA‐MS) to evaluate the stability of the sulfate groups. With the signals of a m/z of 64 and 80, the release of SO_2_ and SO_3_ from the materials was measured (Figure S13). For all the materials analysed, no significant signal was observed over the whole temperature range. The m/z signal of 64 and 80 remained similar during heating of the material. In combination with no significant weight loss, the sulfated surface groups of the mesoporous catalysts showed to be relatively stable at reaction temperatures.

The products of the pyrolysis reactions with the SZ‐20 and SZ‐50 catalyst materials with the lowest and highest weight loading of Zr(SO_4_)_2_ were analysed to whether sulfur is released from the catalyst during the pyrolysis. After the pyrolysis with the low *M*_w_ PE, 104 and 70 ppm of sulfur was present for SZ‐20 and SZ‐50, respectively, while with the high *M*_w_ PE, 159 and 93 ppm of sulfur was present in the oil. More sulfur is released during the reaction with the lowest weight loading of Zr(SO_4_)_2_. While the presence of sulfur is undesirable in pyrolysis oils, the levels are below the industrial threshold of 500 ppm.^[46]^

### Catalyst Reusability

**Figure 8:**
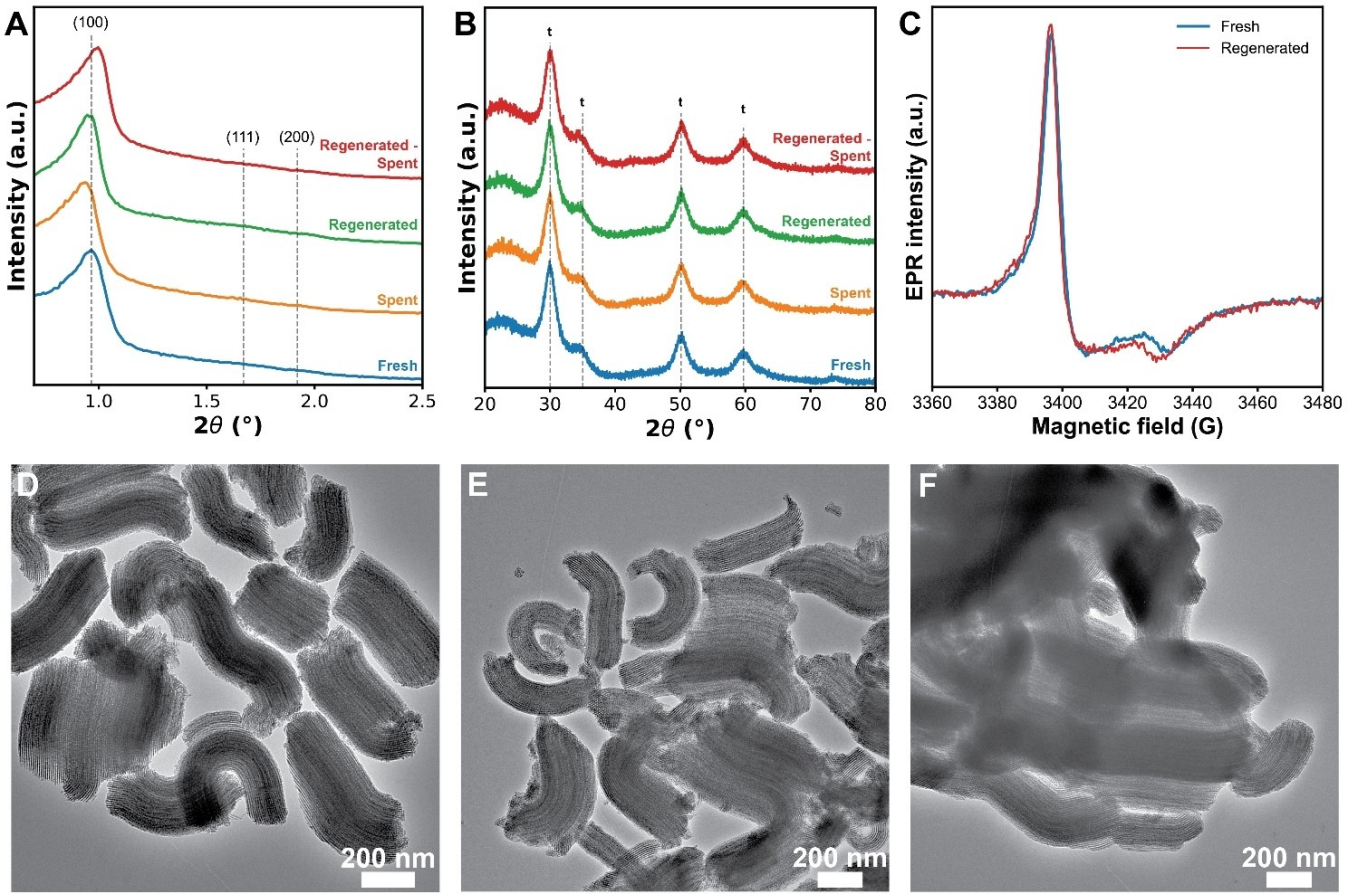
Overview of the properties of the SZ‐50 catalyst used to showcase the reusability of the materials under study. X‐ray diffraction (XRD) patterns at (a) small 2θ angles with annotations to the (100), (111) and (200) reflections of SBA‐15, and (b) larger 2θ angles with annotations to the peaks corresponding to tetragonal zirconia. (c) Mass‐normalized electron paramagnetic resonance (EPR) spectra of the fresh and regenerated material. Transmission electron microscopy (TEM) images of (d) spent, (e) regenerated, and (f) regenerated‐spent material.

To test the potential reuse of the mesoporous catalyst, one of the materials (SZ‐50 used with low *M*_w_ PE) was regenerated by removal of the coke deposition at 400 °C in air and denoted ‘regenerated’. The regenerated catalyst was subsequently used for the batch catalytic conversion of low *M*_w_ PE and denoted ‘regenerated‐spent’. Analysis of the fresh, spent, regenerated and regenerated‐spent catalyst showed no significant changes in XRD, indicating that the ordered mesoporosity and zirconia crystal structure were stable over a regeneration cycle (Figure 8A/B). Spent samples with other sulfated zirconia loadings also showed no significant changes (Figure S15–16). TEM of the SZ‐50 series showed the presence of the ordered mesopores of SBA‐15 after all treatments (Figure 8D–F). EPR showed the presence of the active Zr^3+^ species upon regeneration (Figure 8C).

**Figure 9:**
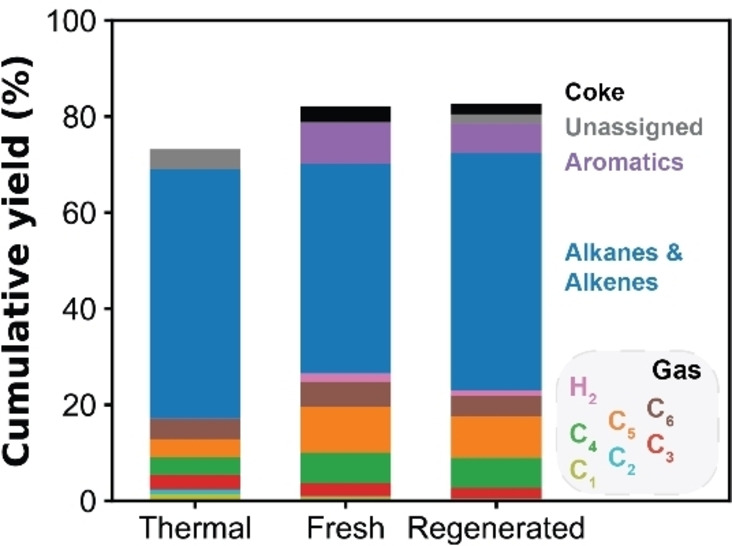
Product distributions of the pyrolysis reaction of low *M*_w_ polyethylene (PE) (4,000 g/mol) with fresh and regenerated SZ‐50 catalyst.

This explains, why the regenerated SZ‐50 catalyst material retains most of its activity in the batch pyrolysis of low *M*_w_ PE (Figure 9). The regenerated catalyst, however, produced less gaseous and more liquid hydrocarbons, with an increase in alkanes/alkenes and a decrease in aromatics in the liquid phase. Furthermore, the coke content on the catalyst material was 2.1 wt %, which is lower than the 3.3 wt % observed on the fresh catalyst material. These changes in products showed that the regenerated material is less active compared to the fresh material, as a higher activity leads to an increase in smaller hydrocarbons, aromatic compounds and coke species. The loss in activity was also observed with TGA, where the degradation temperature of PE was 30 °C higher compared to the fresh catalyst (Figure S20). Nevertheless, these findings show the ability of the S‐ZrO_2_/SBA‐15 material to be reused in the pyrolysis of PE.

### Mechanistic Understanding

**Scheme 2:**
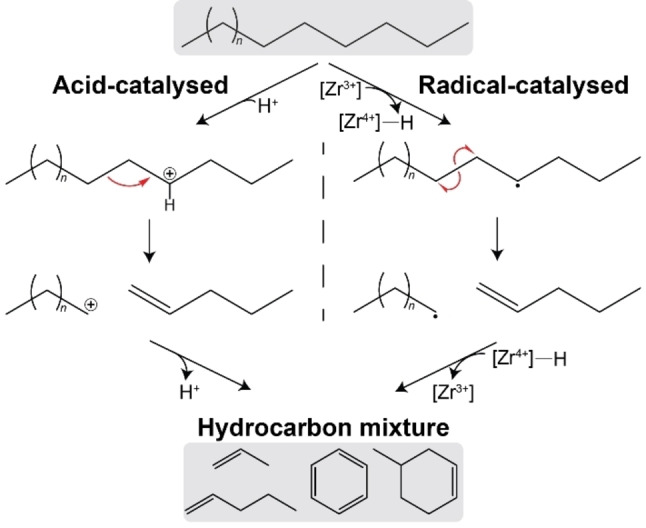
Mechanistic overview of acid‐ and radical‐catalysed degradation of polyethylene with the use of the S‐ZrO_2_/SBA‐15 catalyst.

We initially hypothesized that the S‐ZrO_2_/SBA‐15 catalyst catalyses the degradation via Brønsted acid sites of the material (left, Scheme 2). The PE will be protonated to form a carbonium ion. This can undergo β–scission to produce smaller fragments, consisting of an unsaturated and a positively charged hydrocarbon. This can further undergo cracking, isomerisation, cyclisation and aromatisation, and be terminated after donation of the proton to give a mixture of hydrocarbons.^[47]^ However, the trend of increasing activity with sulfated zirconia loading cannot be explained by the virtually equal acidity observed with ammonia TPD and pyridine FTIR (Table 1).

We therefore investigated the possibility of an alternative mechanism (right, Scheme 2), in which the degradation can be catalysed by the Zr^3+^, which we observed with EPR. The Zr^3+^ can cleave a hydrogen off the PE backbone and form a Zr^4+^–H complex and a hydrocarbon radical.^[35]^ This radical can undergo homolytic cleavage to produce smaller fragments, an unsaturated and a hydrocarbon radical. The radical can undergo further reaction, similar to acid‐catalysed cracking, and be terminated after donation of the hydrogen from the Zr^4+^–H complex to generate a hydrocarbon mixture and regenerate the Zr^3+^.

## Conclusions

A mesoporous sulfated zirconia‐containing SBA‐15 (S‐ZrO_2_/SBA‐15) was prepared to assess the influence of active site density and accessibility on the catalytic pyrolysis of polyethylene (PE) and was compared to a series of microporous zeolite Y materials. *In situ* X–ray diffraction (XRD) measurements showed that low *M*_w_ PE (4,000 g/mol) is able to enter the mesopores of SBA‐15 and lifts the accessibility limitations which were observed for microporous materials. However, a limit of accessibility is reached for a high *M*_w_ PE (350,000 g/mol).

In catalyst activity measurements of S‐ZrO_2_/SBA‐15, the degradation temperature of low *M*_w_ PE correlates with active site density, while this was not the case for zeolite Y. This shows that the S‐ZrO_2_/SBA‐15 material could be used to assess the intrinsic effect of active sites on activity.

All catalysts produced products with a higher carbon number compared to thermal pyrolysis, with mostly alkanes/alkenes in the liquid phase and almost no C_1_–C_2_ products formed. The sulfated zirconia loading did not influence the product distribution over the S‐ZrO_2_/SBA‐15 catalysts. However, compared to zeolite Y catalysts, the selectivity towards aromatic compounds was lower. Similarly, the amount of coke deposits over S‐ZrO_2_/SBA‐15 materials was lower and could be removed at lower temperatures. This is a desired effect, although it indicates an overall lower activity of S‐ZrO_2_/SBA‐15 compared to zeolite Y. S‐ZrO_2_/SBA‐15 converted the PE materials towards a useable product mixture and requires milder temperatures for regeneration compared to zeolite Y. The activity of S‐ZrO_2_/SBA‐15 was partially retained after regeneration and the material remained structurally intact.

However, the catalytic mechanism proceeded differently than expected, not mainly via an acid‐based mechanism, but was linked to Zr^3+^ species on the catalyst. We propose that both acid‐based as well as radical‐based pathways play a role, while the latter dominates.

## Conflict of Interests

The authors declare no conflict of interest.

1

## Supporting information

As a service to our authors and readers, this journal provides supporting information supplied by the authors. Such materials are peer reviewed and may be re‐organized for online delivery, but are not copy‐edited or typeset. Technical support issues arising from supporting information (other than missing files) should be addressed to the authors.

Supporting Information

## Data Availability

All data and python scripts utilized in the manuscript have been uploaded to the Yoda repository and are available under 10.24416/UU01‐8GB6YL.
