# Supplementary material for: Catalytic Pyrolysis of Polyethylene with Microporous and Mesoporous Materials: Assessing Performance and Mechanistic Understanding
Source: ChemSusChem. 2024 Nov 7;18(7):e202401141. doi: 10.1002/cssc.202401141 (PMC11960579; doi:10.1002/cssc.202401141)
Supplement: Supplementary file 1 — Supporting Information [file CSSC-18-e202401141-s001.pdf]

ChemSusChem

Supporting Information

Catalytic Pyrolysis of Polyethylene with Microporous and Mesoporous Materials: Assessing Performance and Mechanistic Understanding

Johan H. van de Minkelis, Adrian H. Hergesell, Jan C. van der Waal, Rinke M. Altink, Ina Vollmer,* and Bert M. Weckhuysen*

Supporting information

1. Materials and methods

1. Materials synthesis

The Santa Barbara Amorphous-15 (SBA-15) material was synthesized using the triblock copolymer poly(ethylene glycol)-*block*-poly(propylene glycol)-*block*-poly(ethylene glycol) (Pluronic P123, Sigma Aldrich) as template. A single batch of SBA-15 material was used for the deposition of sulphated zirconia (S-ZrO_2) in the second phase. 18 g of P123 was dissolved in 540 ml ultrapure water (Milli-Q) and 90 ml HCl (37 wt%, Merck), while stirring at 300 rpm. Tetraethyl orthosilicate (TEOS, 98%, Merck) was used as silica source. 42 ml of TEOS was added to the mixture. The mixture was aged at 40 °C for 20 h, while stirring at 300 rpm. Afterwards the mixture was further aged at 100 °C for 24 h. The mixture was filtered under vacuum and washed with additional ultrapure water, until the filtrate had a neutral pH. The residue was dried at 120 °C for 12 h and calcined at 650 °C for 5 h with a ramp of 2.5 °C/min.

A series of sulphated zirconia on SBA-15 materials were synthesized by an incipient wetness impregnation (IWI) method using $Zr(SO_4)_2 \cdot 4H_2O$ (98+%, Sigma Aldrich). The materials were prepared with different weight loadings of $Zr(SO_4)$, namely 25, 33, 43 and 50 wt%. For these materials, 0.47 g, 0.93 g, 1.40 g and 1.87 g $Zr(SO_4)_2 \cdot 4H_2O$ were used, respectively. The $Zr(SO_4)_2 \cdot 4H_2O$ salt was dissolved in 1.2 ml ultrapure water. The solution was used for impregnation of 1.5 g SBA-15. The impregnated SBA-15 was dried at 120 °C for 12 h and calcined at 650 °C for 5 h with a ramp of 2.5 °C/min.

As microporous catalyst material, a series of commercially available zeolite Y materials (Zeolyst) were used. These materials had a SiO_2/Al_2O_3 of 12 (CBV 712), 30 (CBV 720), 60 (CBV 760), and 80 (CBV 780).

2. Materials characterization

X-ray diffraction (XRD) patterns were collected with a Bruker D2 Phaser using a Cu source (0.154184 nm). XRD patterns of the SBA-15 materials were collected in the 2θ 0.7 to 5° region with a 0.1 mm slit, a 0.5 mm anti-scatter knife, a position sensitive detector (PSD) opening of 1°, no sample rotation and a degree increment of 0.01°. The XRD patterns of ZrO_2 were collected in the 2θ 15 to 80° region with a 1.0 mm slit, a 2.0 mm anti-scatter knife, a PSD of 3°, a sample rotation of 15 rpm and a degree increment of 0.02°. Both regions were measured with an integration time of 0.5 s.

Nitrogen adsorption and desorption measurements were performed on a 3P Instruments Sync 400 at −196 °C. The samples were outgassed at 150 °C for 10 h under vacuum. The specific surface area was calculated with the Brunauer–Emmett-Teller (BET) method. Pore volume was determined using the Gurvich equation.

Transmission electron microscopy (TEM) measurements were performed on a Tecnai F20 microscope at 200 kV. Elemental mapping was performed using HAADF-STEM-EDX on a TFS Talos F200x at 200 kV.

The total acidity of the materials was determined with ammonia temperature programmed desorption (NH_3-TPD). The NH_3-TPD measurements were carried out on a Micromeritics AutoChem II instrument. Typically, 100 mg of sample was dried at 400 °C for 30 min with a temperature ramp of 5 °C/min under a He flow. Afterwards, the sample was cooled down to 100 °C and NH_3 adsorption was performed by pulsing 25 cm^3 NH_3 per minute. Desorption was performed by increasing the temperature to 700 °C with a temperature ramp of 5 °C/min.

The different types of acid sites were examined with Fourier-transform infrared (Py-FT-IR) spectroscopy in combination with pyridine as probe molecule. Measurements were carried out on a Thermo Scientific Nicolet iS5 spectrometer. 10 mg of sample was pelletized at 2 tons into a pellet with a 13 mm diameter. The sample was dried *in situ* under vacuum (10^{-5}-10^{-6} bar) at 550 °C for 1 h with a temperature ramp of 5 °C/min. Subsequently, the sample was cooled down to 40 °C and 12 mbar of pyridine was dosed for 30 min. The excess of pyridine was removed by evacuation under vacuum and heated to 150 °C for

30 min with a 10 °C/min temperature ramp. Pyridine desorption was performed at 550 °C with a temperature ramp of 10 °C/min.

Electron paramagnetic resonance (EPR) spectroscopy was performed on a Bruker EMXplus spectrometer. The EPR measurements were performed in a continuous wave mode at 99 K, a microwave frequency of 9.41 GHz, a modulation amplitude of 1 G, and a modulation frequency of 100 kHz. Typically, 30 to 60 mg of sample was loaded into an EPR tube. The EPR spectra are normalized by the sample mass, with the relative Zr^{3+} concentrations being calculated by a double integration of the EPR spectra between 3300 and 3500 G.

3. Pore accessibility

In situ XRD patterns were collected with a Bruker D2 Phaser using a Cu source (0.154184 nm). A XRK 900 (Anton Paar) reactor chamber was used for measurements at elevated temperatures. A 2:1 PE-to-SBA-15 ratio was used. A scatter knife of 0.5 mm was used on top of the sample holder that was located in the XRK 900 reactor chamber. The z-axis of the sample was aligned to position the peak of the (100) reflection at same 2θ values for all samples. Patterns were collected in the 2θ 0.7 to 5° region with a 0.1 mm slit, an empty slit box, a position sensitive detector (PSD) opening of 1°, a degree increment of 0.01°. Patterns were collected every 99.9 s with a 60 s dwell afterwards. The sample was heated from 25 °C with a 1 °C/min ramp to 150 °C with a hold that contained 5 measurements. Afterwards the sample was cooled down to 35°C with a -1 °C/min ramp. This was all done in a 100 ml/min N_2 atmosphere.

4. Catalytic performance

Catalytic performance was assessed by thermogravimetric analysis (TGA) and was carried out on a PerkinElmer TGA 8000 instrument. The TGA measurements were performed using two different polyethylene (PE) materials, namely a low molecular weight (M_w) PE of 4,000 g/mol with a number average molar weight (M_n) of 1,700 g/mol (Sigma Aldrich) and a high M_w PE of 350,000 g/mol with a M_n of 13,000 g/mol (Sabic). Typically, the total sample weight was ~ 5 mg. For thermal degradation, the sample consisted of only PE, while for catalytic degradation, the sample consisted of a mixture of PE and catalyst material in a 1:1 ratio. TGA was performed at 600 °C for 5 min with a temperature ramp of 10 °C/min under a N_2 flow of 45 ml/min. Afterwards, the sample was cooled down to 50 °C and the flow was switched to 45 ml/min O_2 for coke burn off. This was carried out at 1000 °C for 5 min with a temperature ramp of 20 °C/min.

Pyrolysis experiments of the low and high M_w PE were carried out in a 50 ml 4590 series Parr autoclave connected to two glass condensers, which were located in an ice bath (Figure S1). The outlet of the second condenser was connected to a gas chromatograph (GC) (TraceGC 1300), which is a GC coupled with to a TCD, FID and mass spectrometer (MS). The samples were collected in 16 sample loops of 100 μl. To quantify H_2, N_2, O_2, methane, ethane, propane and propene, a Rt-Q-Bond 15m*0.32mm, a Rt-Q-Bond 30m*0.32mm and a Molsieve 5A 30m*0.32mm column and an TCD detector was used. To quantify the hydrocarbons, a Rxi-5ms, 30m, 0.32mm ID, 0.5 μm column and an FID detector was used. To identify the hydrocarbons, the sample was transferred to a ISQ 7000 series mass spectrum after passing the Rxi-5ms, 30m, 0.32mm ID, 0.5 μm column.

For the pyrolysis experiments, the autoclave was loaded with 1 g of PE and 0.5 g of catalyst material. The whole setup was under a N_2 flow of 50 ml/min. Pyrolysis was carried out at 400 °C for 1 h with a temperature ramp of 10 °C/min. Samples were collected every 6 min starting after 10 min of heating. Afterwards, the liquid product was collected from the condensers and weighed and the spent catalyst was recovered from the autoclave. The liquid product was analysed by GCxGC (Shimadzu, GC-2010 Plus) coupled with an FID and MS (Shimadzu, GCMS-QP2010 Ultra). 0.5 μl of sample was used as injection volume. The samples were separated using a GC 60m, 0.25 mm ID, 0.25 μm column and a VF-5ms, 30 m, 0.25 mm ID, 0.25 μm column. Column oven temperature was increased from 40 °C to 280 °C with a 2.50 °C/min ramp and a 5 min holding time at begin and end temperature. The coke content was determined by burning off the carbon species by TGA at 1000 °C for 5 min with a temperature ramp of 20 °C/min with a 45 ml/min O_2 flow.

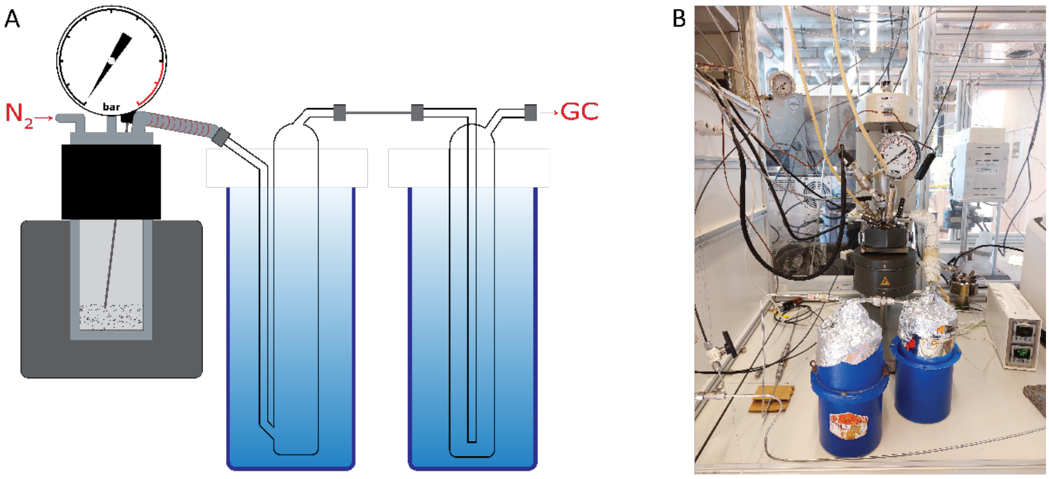

Figure S1: Autoclave reactor used for the pyrolysis of polyethylene. (a) Schematic and (b) actual setup.

5. Catalyst stability

Catalyst stability was determined by thermogravimetric analysis (TGA, PerkinElmer TGA 8000) coupled with mass spectrometry (MS, Hiden Analytical). Pure catalyst material was subjected towards a temperature program to measure the release of sulfate groups. The samples were heated from 30 °C to 1100 °C with a 10 °C/min ramp under a 25.0 ml/min argon flow. With the mass spectrometer, scans were taken at m/z 18, 32, 40, 48, 64 and 80 during the heating of the sample to detect the release of certain species.

6. Catalyst regeneration

The capability of catalyst regeneration was determined by removing of the carbon deposits from the spent SZ-50 catalyst which was used in the pyrolysis reaction with low M_w PE. The material was regenerated by heating it to 400 °C with a 10°C/min ramp for 5 h. The regenerated material was afterwards used in the batch pyrolysis reaction of low M_w PE. The amount of PE was scaled to the amount of catalyst material, with a 2:1 ratio. The batch pyrolysis was performed according to the procedure in section 1.4.

2. X-ray diffractograms

The synthesized SBA-15 and S-ZrO_2/SBA-15 materials were analysed by X-ray diffraction (XRD) to confirm the formation of the desired materials structures. Small-angle XRD was used to confirm the SBA-15 structure, while XRD at larger 2θ angles were used to confirm the presence of ZrO_2. Small-angle XRD showed three peaks, corresponding to the d_{100}, d_{110} and d_{200} planes of the SBA-15 material.[44] The intensity of the three XRD peaks decreases with increasing S-ZrO_2 wt%, indicating that the S-ZrO_2 introduced partial structural collapse, which makes the structure less ordered. Based on the d_{100} peak and using the Bragg equation, the lattice spacing between the mesoporous were determined. For all synthesized materials under study, the lattice distance was between 10.5 and 11.1 nm. This showed that all materials have approximately the same hexagonal mesoporous structure.

The XRD measurements at larger 2θ angles showed that ZrO_2 was present for all synthesised SBA-15 materials under study. The materials showed four distinctive peaks that correspond to a tetragonal ZrO_2 phase.[48] Additionally, the XRD peak intensity increased with increasing weight loading of S-ZrO_2, indicating that the crystallite size increased. The crystallite size was determined using the Scherrer equation and based on the FWHM of the peak at 2θ 50°. The crystallite size increased from 3.2 nm up to 3.9 nm based on the 33, 43 and 50 wt% S-ZrO_2/SBA-15 catalyst materials. The signal of the 20 wt% material was not sufficiently intense to determine the crystallite size. The determined crystallite sizes are smaller than the 5 nm pores of SBA-15. This indicates that the S-ZrO_2 crystallites and therefore the

active sites could be present inside the mesopores, although we cannot rule out that they are partially covering the outer surface of the SBA-15 materials.

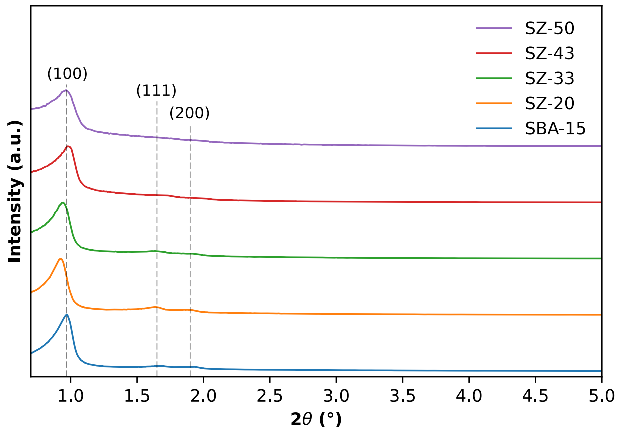

Figure S2. X-ray diffractograms (XRD) of SBA-15 and the four synthesised S-ZrO_2/SBA-15 catalyst materials in the small 2θ angle region. Reflections of (100), (110) and (200) of SBA-15 are indicated.

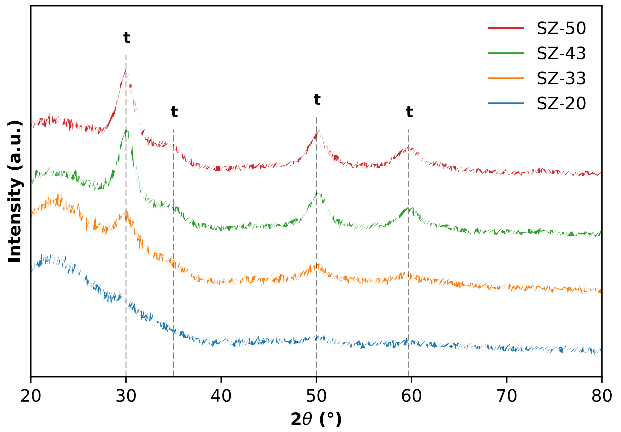

Figure S3. X-ray diffractograms (XRD) of the four synthesised S-ZrO_2/SBA-15 catalyst materials in the large 2θ angle region. Reflections of tetragonal (t) zirconia are indicated.

3. N_2 physisorption isotherms

The pore structures of the synthesised materials were investigated by nitrogen physisorption. N_2-physisorption showed isotherms (Figure S4) that correspond to a type IV isotherm, defined by IUPAC classification, which is typical for a mesoporous material.[49] The catalyst material showed a change in hysteresis loop with increasing weight loading. Both the SBA-15, the 20 and 43 wt% S-ZrO_2/SBA-15 catalyst showed a type H1 hysteresis loop, which corresponds to a narrow range of uniform mesoporous. However, the 33 and 50 wt% catalyst materials showed a H5 hysteresis loop, which indicates that these catalyst materials consist of (partially) blocked mesopores. This could be the result of large S-ZrO_2 particles inside the pores of the SBA-15 material. The BET surface and total pore volume showed a trend corresponding to the increase of S-ZrO_2. As-synthesised SBA-15 had a BET surface area of 650 m^2/g and a pore volume of 1.0 cm^3/g. Introducing S-ZrO_2 in SBA-15 decreased the BET surface area to 330 m^2/g and the pore volume to 0.6 cm^3/g, showing that the S-ZrO_2 particles are present inside the mesoporous, partially blocking them.

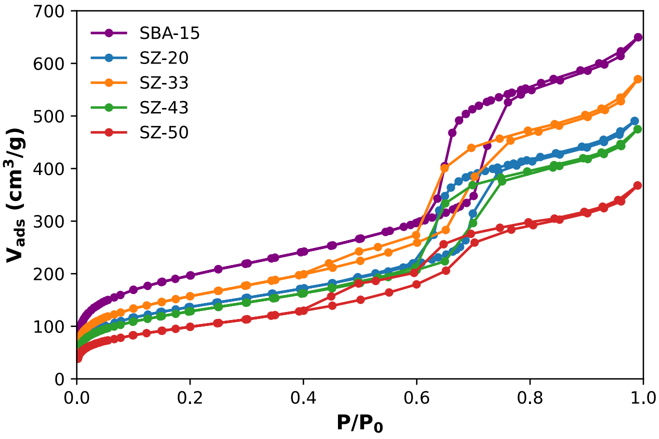

Figure S4: The N_2-physisorption isotherms of SBA-15 and the four S-ZrO_2/SBA-15 catalyst materials under study.

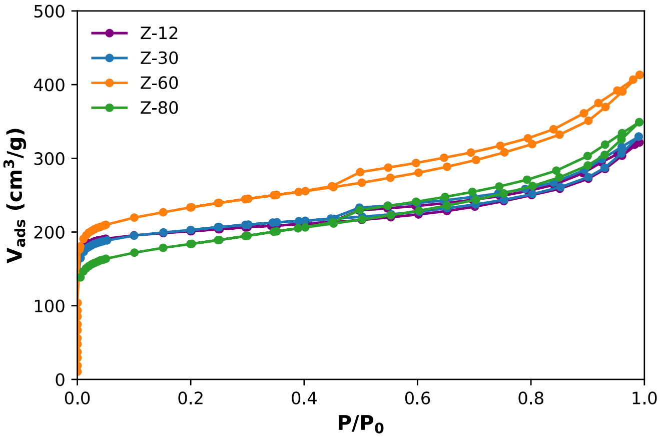

Figure S5: The N_2-physisorption isotherms of the four zeolite Y materials under study.

4. Electron microscopy

To further confirm the formation of the desired structures, the materials were analysed by transmission electron microscopy (TEM). The obtained TEM images, shown in Figure S5, clearly reveal the ordered mesopores of SBA-15. The SBA-15 material appeared as aggregated rodlike crystals. After impregnation of $Zr(SO_4)_2{\cdot}4H_2O$ and calcination, the ordered mesopores remained for all four synthesised catalyst materials. This showed that we are capable of creating a series of catalyst materials with a similar mesoporous structure similar, while we can control the weight loading of S-ZrO_2. Next, we investigated whether this also leads to the desired control over the acidity.

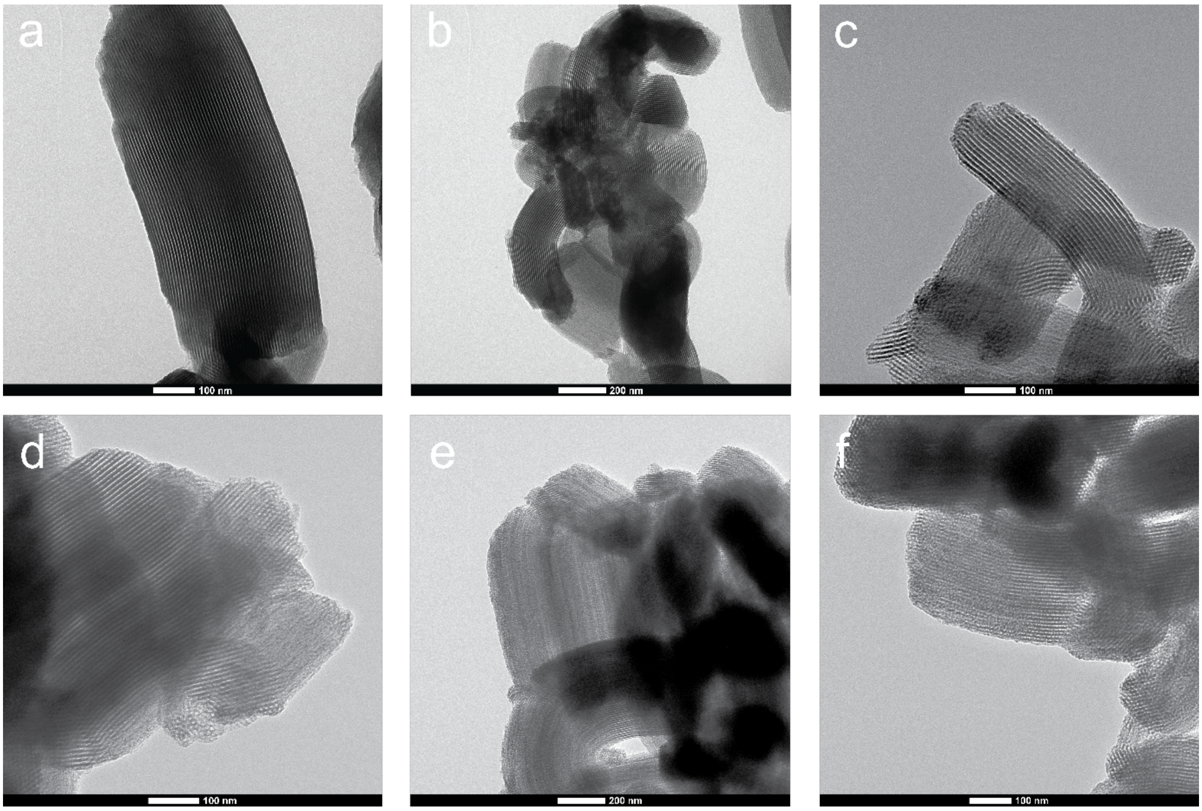

Figure S6: Transmission electron microscopy (TEM) images of (a,b) SBA-15, (c) SZ-20, (d) SZ-33, (e) SZ-43 and (f) SZ-50.

5. NH_3-temperature programmed desorption

NH_3-temperature programmed desorption (TPD) was used to investigate the total acidity of the synthesised catalyst materials and the reference catalyst zeolite Y. The NH_3-TPD measurements showed a clear difference between the S-ZrO_2/SBA-15 catalysts and zeolite Y, which is used as reference material. While the zeolite has three TPD peaks distributed over the whole temperature range, indicating weak (190 °C), medium (350 °C) and strong (500 °C) acid sites, the S-ZrO_2/SBA-15 catalyst materials only show a broad peak in the range of 180 – 350 °C (Figure S6). This observation indicates that these catalyst materials contain a mixture of weak and medium strength acid sites. Additionally, a signal is observed at higher temperature ranges (400 – 700 °C). This could be the indication of a stronger acid site, however, the signal is not observed for all weight loadings and different batches of the S-ZrO_2/SBA-15 materials. It is possible that the release of sulphate groups (SO_2 and SO_3) from the catalyst surface are showing up as a signal on the TCD.

As the signal at higher temperatures corresponds to the release of sulphate groups and not the desorption of ammonia, the total amount of acid sites is determined in the 100 – 400 °C temperature region for the S-ZrO_2/SBA-15 materials. From this, the total acid site concentration was between 0.16 and 0.19 mmol g^{-1} for the catalyst. Compared to the reference zeolite Y catalyst materials, the S-ZrO_2/SBA-15 catalysts all showed a lower total acidity (Figure S7).

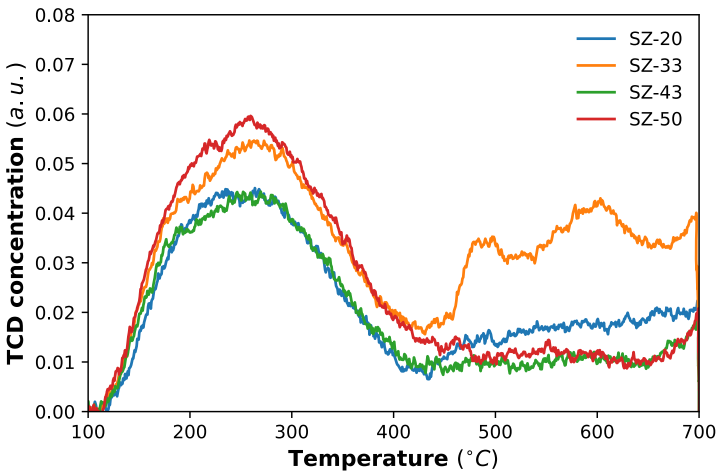

Figure S7: NH_3 temperature programmed desorption (NH_3-TPD) profiles of the four synthesized S-ZrO_2/SBA-15 catalyst materials.

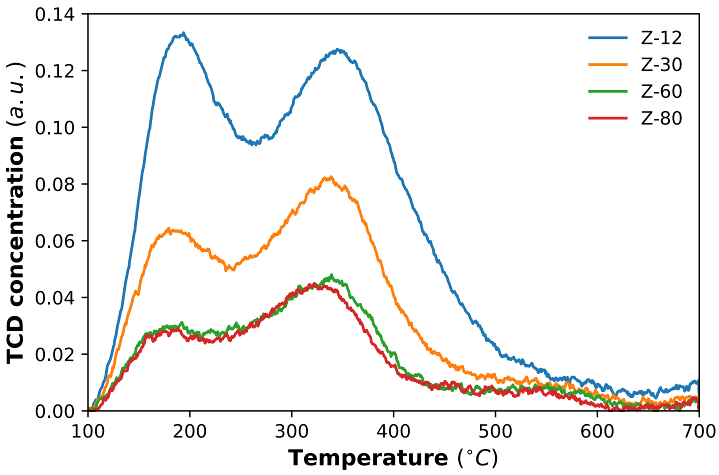

Figure S8: NH_3 temperature programmed desorption (NH_3-TPD) profiles of the four zeolite Y catalyst materials.

6. Pyridine Fourier transform-infrared spectroscopy

Pyridine-FT-IR spectroscopy was utilized to determine the relative ratio between the number of Brønsted (BA) and Lewis acid (LA) sites. Adsorbed pyridine molecules are known to have different vibrational frequencies in the infrared region of the spectrum, depending on the nature and type of acid site, which it is adsorbed to. For pyridine bound to Brønsted acid sites, spectroscopic features will become visible at 1545 and 1640 cm^{-1}, while for pyridine bound to Lewis acid site these spectroscopic features will be present at 1455 and 1607 cm^{-1}.[50] A combination band for both type of acid sites is visible at 1495 cm^{-1}. Figure S8 shows an overview of the pyridine-FT-IR spectra of the S-ZrO_2/SBA-15 catalysts. Pyridine-FT-IR spectroscopy showed that the S-ZrO_2/SBA-15 catalysts consist of both Brønsted and Lewis acid sites. The catalyst materials show to have more Lewis acid sites with a BA:LA ratio between 0.16 and 0.21. Changing the weight loading did not have a significant effect to the BA:LA ratio. Compared to the zeolite materials (Figure S9), the BA:LA ratio is significantly different. The zeolite materials have a large presence of Brønsted acid sites.

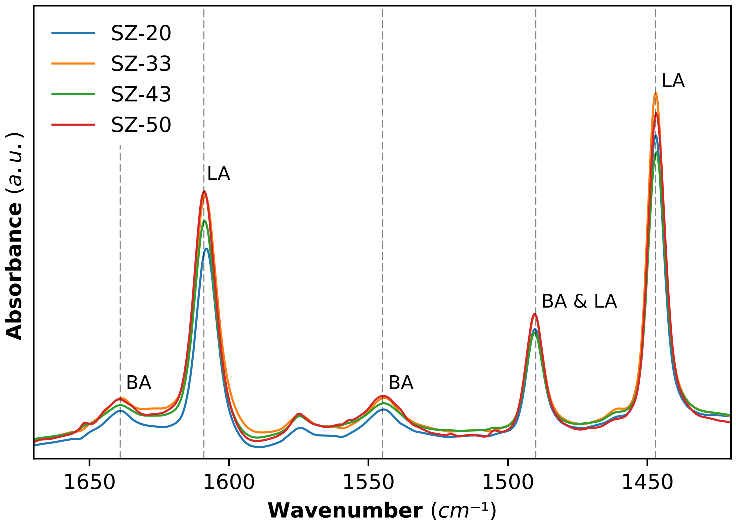

Figure S9: Fourier transform-infrared (FT-IR) spectrum after pyridine adsorption of the four synthesized S-ZrO_2/SBA-15 catalyst materials (SZ-20, 33, 43, and 50).

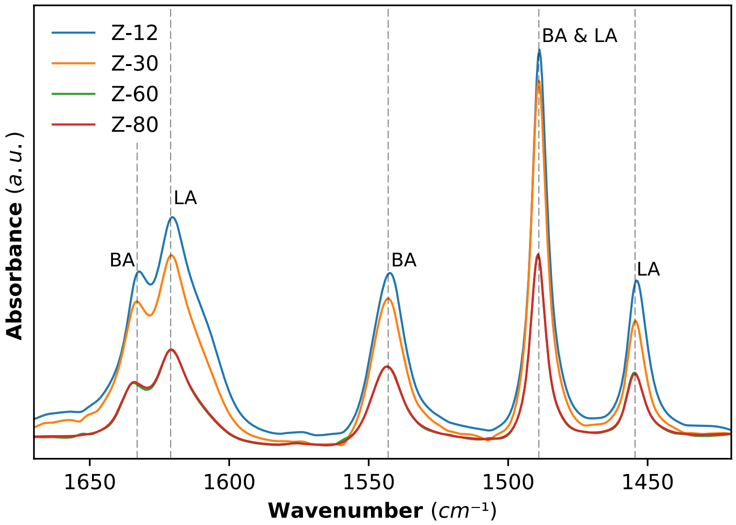

Figure S10: Fourier transform-infrared (FT-IR) spectrum after pyridine adsorption of the four zeolite Y catalyst materials (Z-12, 30, 60, and 80).

7. Thermogravimetric analysis

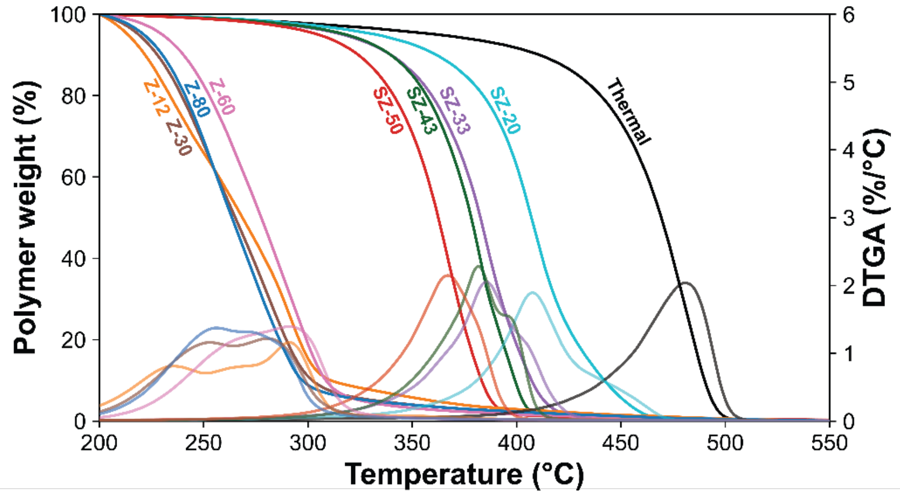

Figure S11: Thermogravimetric analysis (TGA) profiles of low M_w PE with the four synthesized S-ZrO_2/SBA-15 (SZ-20, 33, 43, and 50) and zeolite Y catalyst materials (Z-12, 30, 60, and 80).

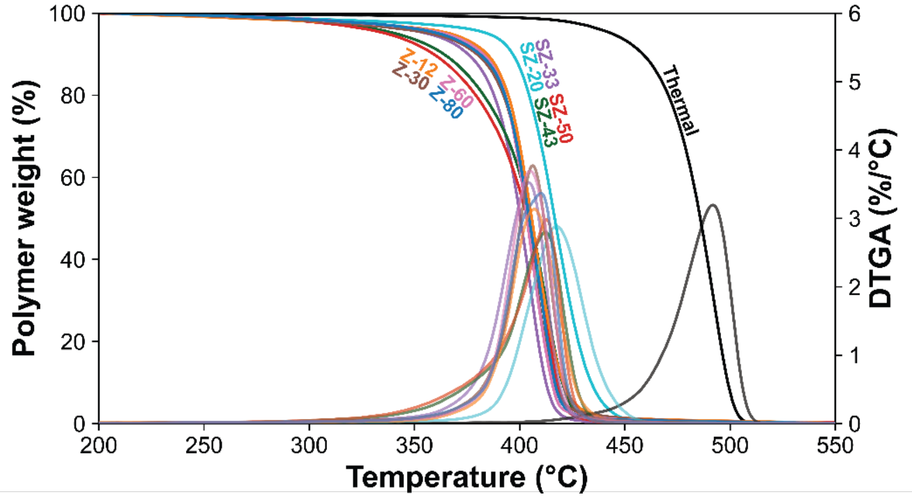

Figure S12: Thermogravimetric analysis (TGA) profiles of high M_w PE with the four synthesized S-ZrO_2/SBA-15 (SZ-20, 33, 43, and 50) and zeolite Y catalyst materials (Z-12, 30, 60, and 80).

8. Radius of gyration

The coil size can be described by the radius of gyration (R_g), which assumes that the polymer forms a gaussian coil and is given by Equation 1.[41]

$$Equation\ (1): R_g = \sqrt{\frac{Nb^2}{6}}$$

with N the number of monomer units and b the statistical segment length (Å). For polyethylene, the statistical segment length is set to be 5.9 Å.[51] For low M_w PE, with a M_n of 1,700 g/mol, the number of monomer units showed to be 61. This results in a radius of gyration of approximately 1.9 nm. For the high M_w PE, with a M_n of 13,000 g/mol and thus 464 monomer units, the radius of gyration for is 5.2 nm.

9. Batch pyrolysis

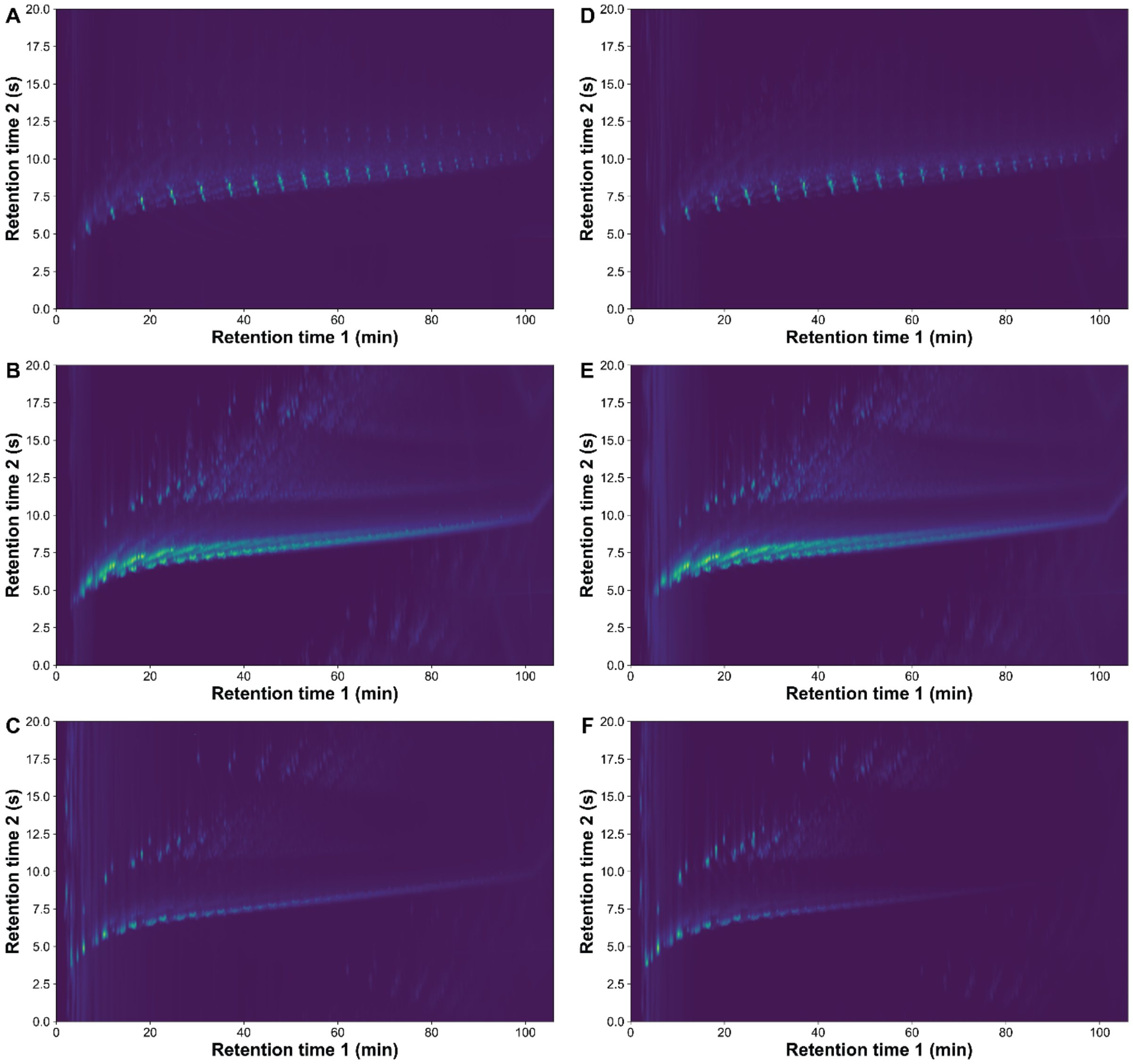

Figure S13: An overview of chromatograms of the liquid products measured with GCxGC collected during the pyrolysis of low M_w PE without catalyst (a), with SZ-50 (b), and Z-12 (c) and of high M_w PE without catalyst (d), with SZ-50 (e), and Z-12 (f).

10. Catalyst stability

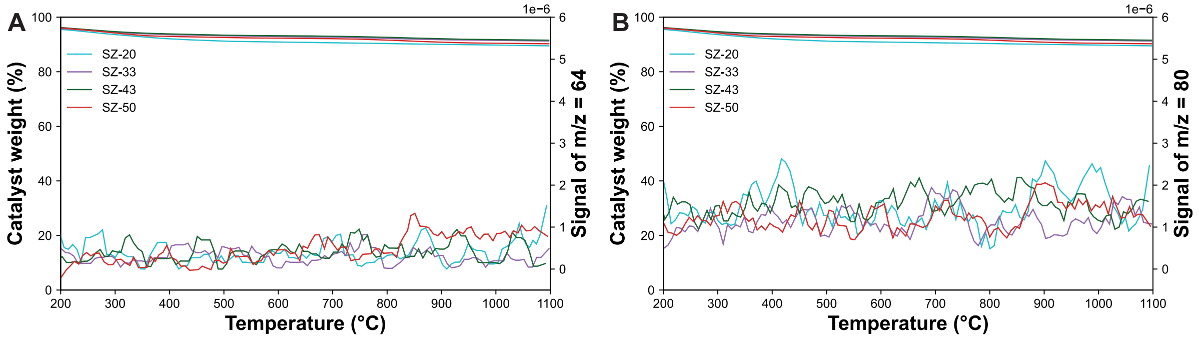

Figure S14: Thermogravimetric analysis (TGA) coupled with mass spectrometry (MS) of the mesoporous catalyst materials based on the release of (a) SO_2 (m/z=64) and (b) SO_3 (m/z=80).

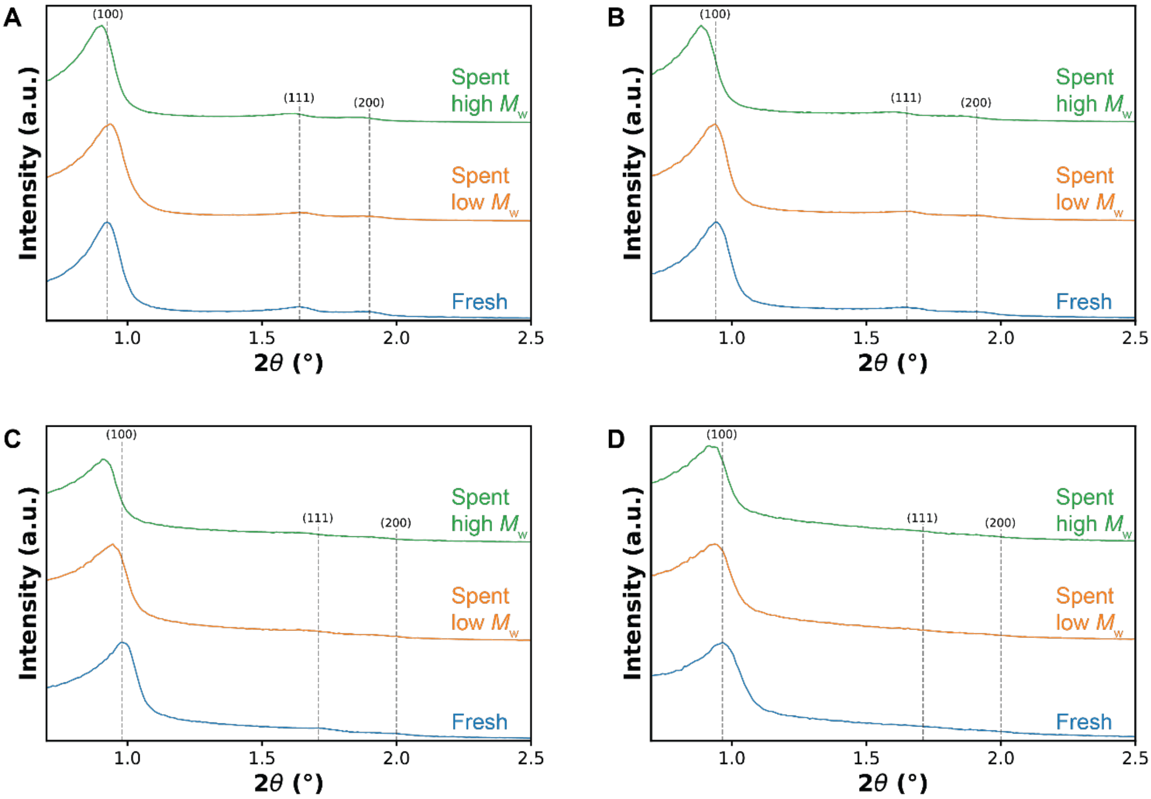

Figure S15: X-ray diffraction (XRD) patterns at smaller angles of the four synthesized S-ZrO_2/SBA-15 materials with (a) 20 wt%, (b) 33 wt%, (c) 43 wt%, and (d) 50 wt% $Zr(SO_4)_2$. The XRD patterns consist of the freshly synthesized materials and the spent materials after the reaction with low and high M_w PE. Reflections of (100), (111) and (200) of SBA-15 are indicated.

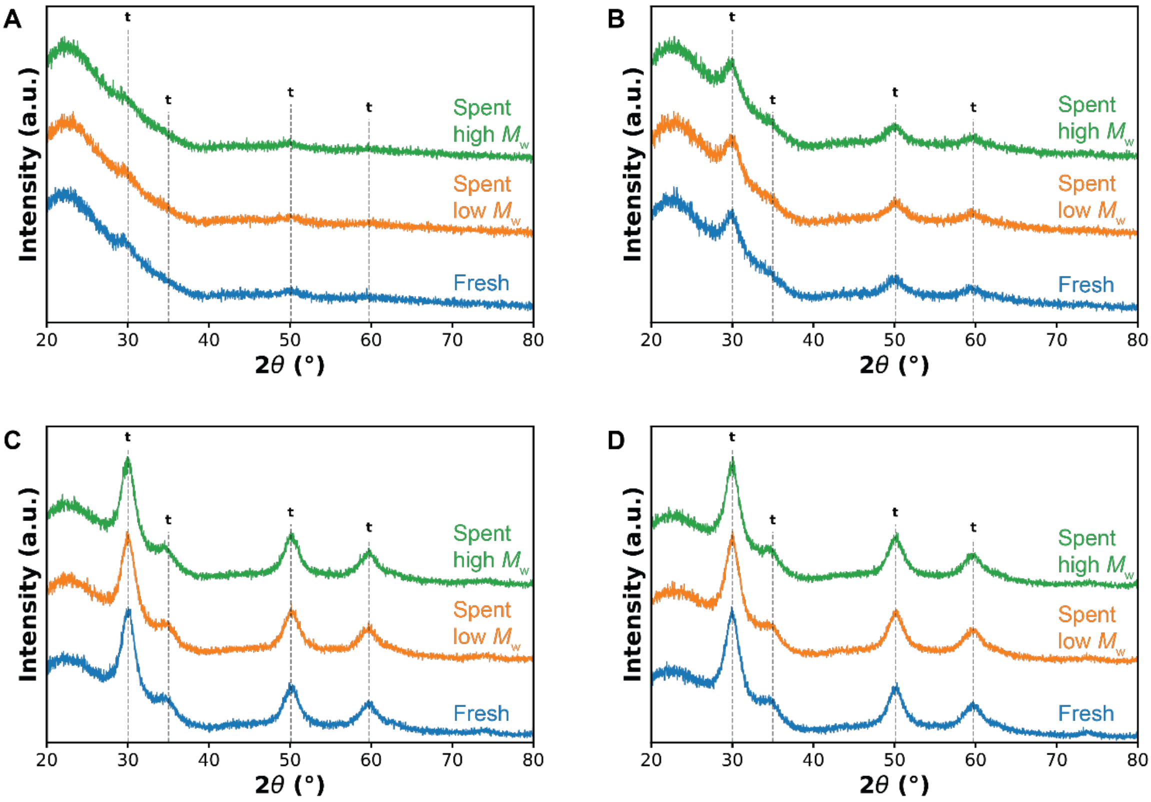

Figure S16: X-ray diffraction (XRD) patterns at larger angles of the four synthesized S-ZrO_2/SBA-15 materials with (a) 20 wt%, (b) 33 wt%, (c) 43 wt%, and (d) 50 wt% $Zr(SO_4)_2$. The XRD patterns consist of the freshly synthesized materials and the spent materials after the reaction with low and high M_w PE. Reflections of tetragonal zirconia are indicated (t).

11. Temperature dependent electron paramagnetic resonance spectroscopy

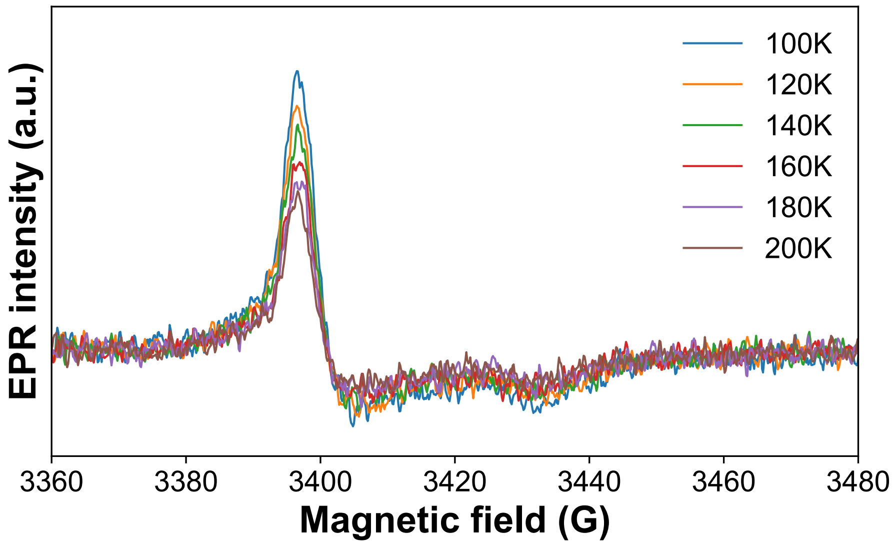

Figure S17. Mass-normalized electron paramagnetic resonance (EPR) spectra of the SZ-50 catalyst material measured at variable temperatures.

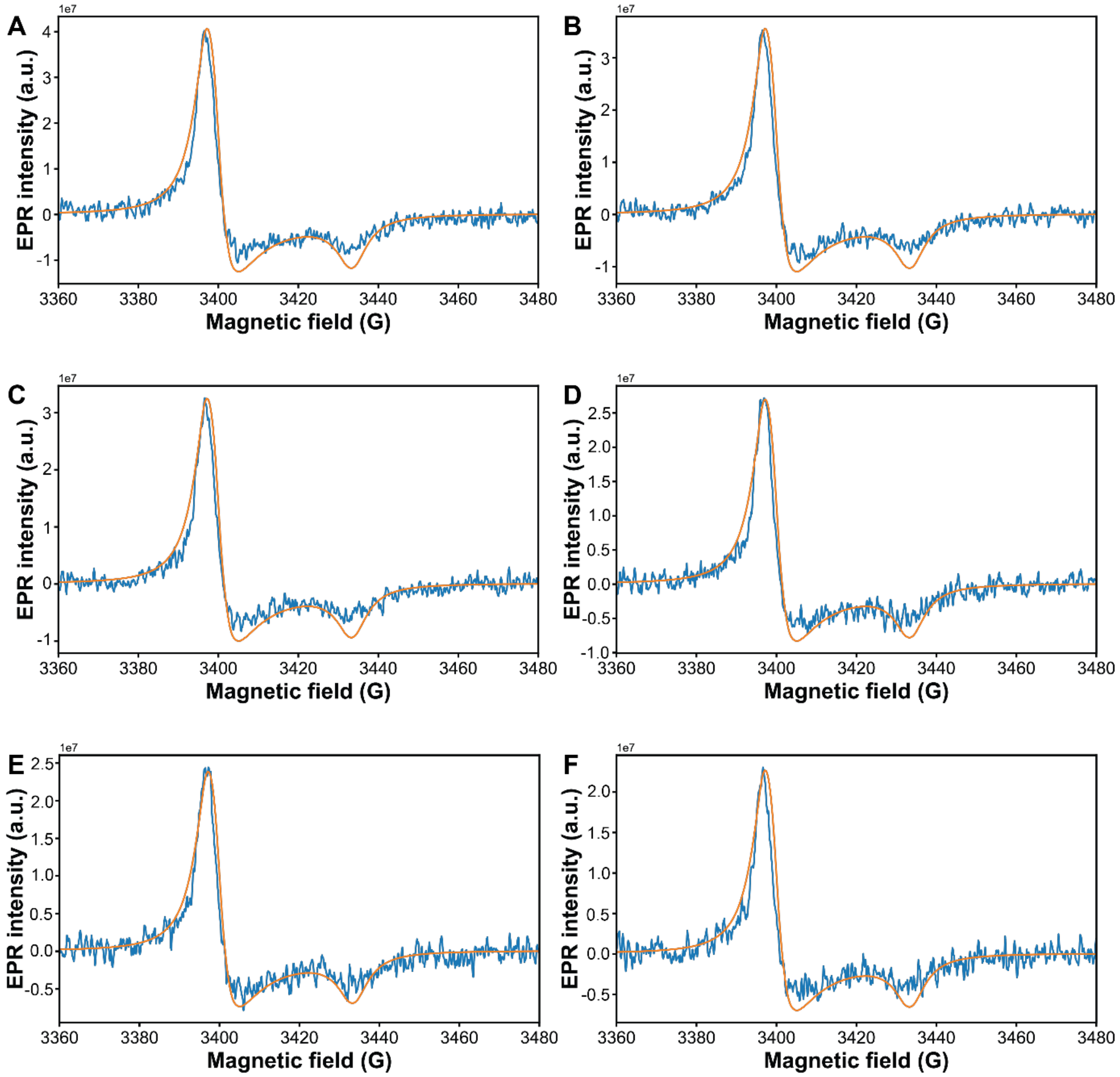

Figure S18. Mass-normalized electron paramagnetic resonance (EPR) spectra of the SZ-50 catalyst material (blue) and simulated spectra (orange) measured at (a) 100 K, (b) 120 K, (c) 140 K, (d) 160 K, (e) 177 K, and (f) 197 K.

12. Simulation of electron paramagnetic resonance spectrum of Zr^{3+}

EPR spectra of $^{90}Zr^{3+}$ were simulated using the EPRsim Python package. A microwave frequency of 9.41 GHz was used, together with a modulation amplitude of 1 G. The spectrum was simulated as solid and axially distorted, with $g_\perp$ = 1.978 and $g_\parallel$ = 1.958, and a Lorentzian line width of 9 G.

Table S1: Experimental, simulated and literature g values for Zr^{3+}.

	$g_x = g_y = g_\perp$	$g_z = g_\parallel$
Experimental	1.978	1.958
Simulated	1.978	1.958
Gionco *et al.*[40]	1.9768	1.9589

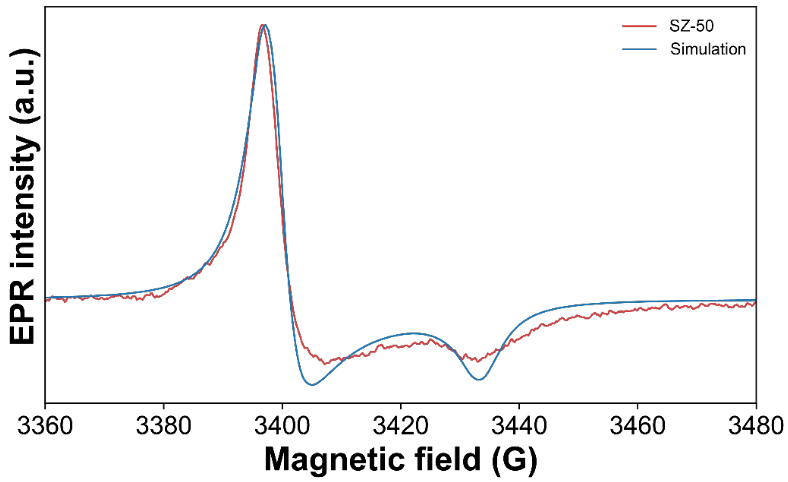

Figure S19. Electron paramagnetic resonance (EPR) spectrum of the SZ-50 catalyst and simulated EPR spectrum of Zr^{3+}.

13. Thermogravimetric analysis with regenerated catalyst

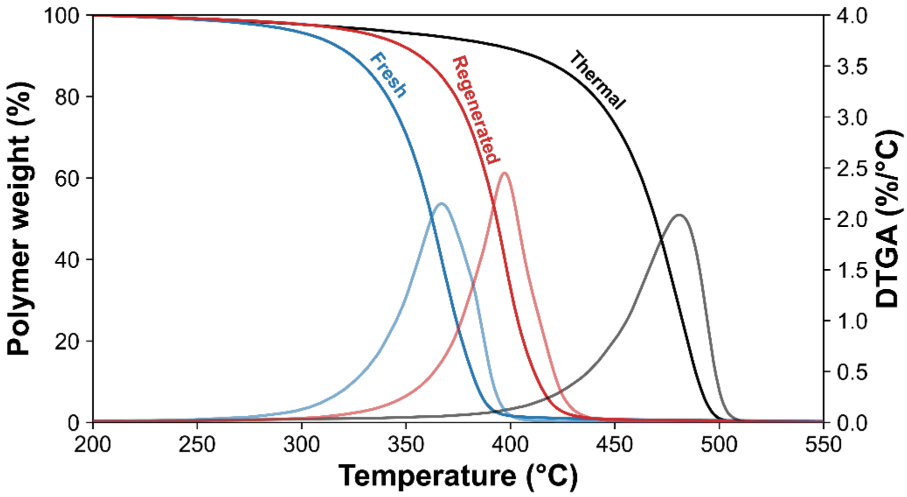

Figure S20: Thermogravimetric analysis (TGA) profiles of low M_w PE with the fresh and regenerated SZ-50 catalyst material.
